# Gender specific airway gene expression in COPD sub-phenotypes supports a role of mitochondria and of different types of leukocytes

**DOI:** 10.1038/s41598-021-91742-x

**Published:** 2021-06-18

**Authors:** Anna Esteve-Codina, Thomas P. Hofer, Dorothe Burggraf, Marion S. Heiss-Neumann, Wolfgang Gesierich, Anne Boland, Robert Olaso, Marie-Therese Bihoreau, Jean-Francois Deleuze, Winfried Moeller, Otmar Schmid, María Soler Artigas, Kathrin Renner, Jens M. Hohlfeld, Tobias Welte, Thomas Fuehner, Lukas Jerrentrup, Andreas Rembert Koczulla, Timm Greulich, Antje Prasse, Joachim Müller-Quernheim, Sumit Gupta, Christopher Brightling, Deepak R. Subramanian, David G. Parr, Umme Kolsum, Vandana Gupta, Imre Barta, Balázs Döme, János Strausz, Mariarita Stendardo, Marco Piattella, Piera Boschetto, Damian Korzybski, Dorota Gorecka, Adam Nowinski, Marc Dabad, Marcos Fernández-Callejo, David Endesfelder, Wolfgang zu Castell, Pieter S. Hiemstra, Per Venge, Elfriede Noessner, Thasso Griebel, Simon Heath, Dave Singh, Ivo Gut, Loems Ziegler-Heitbrock

**Affiliations:** 1 CNAG-CRG, Centre for Genomic Regulation (CRG), Barcelona Institute of Science and Technology (BIST), Barcelona, Spain; 2 Universitat Pompeu Fabra (UPF), Barcelona, Spain; 3 EvA Study Center, Helmholtz Zentrum Muenchen and Asklepios-Klinik, Gauting, Germany; 4 Centre National de Recherche en Génomique Humaine (CNRGH), Institut de Biologie François Jacob, CEA, Université Paris-Saclay, Evry, France; 5 Institute for Lung Biology and Disease, Helmholtz Zentrum Muenchen, Neuherberg-Munich, Germany; 6 Internal Medicine III, University Hospital Regensburg, Regensburg, Germany; 7 Fraunhofer Institute for Toxicology and Experimental Medicine, Hannover, Germany; 8 Department of Respiratory Medicine, Hannover Medical School, Hannover, Germany; 9 Member of the German Center for Lung Research (DZL), Marburg, Germany; 10 Member of the German Center for Lung Research (DZL), Hannover, Germany; 11 Department of Medicine, Pulmonary and Critical Care Medicine, University Medical Center Giessen and Marburg, Philipps-University, Marburg, Germany; 12 Department of Pneumology, Faculty of Medicine, University of Freiburg, Freiburg, Germany; 13 Institute for Lung Health, NIHR Leicester BRC, University of Leicester, Leicester, UK; 14 Department of Respiratory Medicine, University Hospitals Coventry and Warwickshire NHS Trust, Coventry, UK; 15 Medicines Evaluation Unit, University of Manchester, University Hospital of South Manchester Foundations Trust, Manchester, UK; 16 Department of Pathophysiology, National Koranyi Institute for TB and Pulmonology, Budapest, Hungary; 17 Department of Tumorbiology, National Koranyi Institute for TB and Pulmonology, Budapest, Hungary; 18 Department of Pneumology, National Koranyi Institute for TB and Pulmonology, Budapest, Hungary; 19 Department of Medical Sciences, University of Ferrara and Ferrara City Hospital, Ferrara, Italy; 20 2nd Department of Respiratory Medicine, National Institute of Tuberculosis and Lung Diseases, Warsaw, Poland; 21 ICT Department, Helmholtz Zentrum Muenchen, Munich, Germany; 22 Department Strategy & Digitalization, Helmholtz Zentrum Muenchen, Munich, Germany; 23 Faculty of Mathematics, Technical University of Munich, Munich, Germany; 24 Department of Pulmonology, Leiden University Medical Center, Leiden, The Netherlands; 25 Department of Medical Sciences, University of Uppsala, Uppsala, Sweden; 26 Immunoanalytics-Core Facility, Helmholtz Zentrum Muenchen, Munich, Germany; 27 Immunoanalytics-Tissue Control of Immunocytes, Helmholtz Zentrum Muenchen, Munich, Germany

**Keywords:** Immunology, Molecular biology, Medical research, Molecular medicine

## Abstract

Chronic obstructive pulmonary disease (COPD) is a destructive inflammatory disease and the genes expressed within the lung are crucial to its pathophysiology. We have determined the RNAseq transcriptome of bronchial brush cells from 312 stringently defined ex-smoker patients. Compared to healthy controls there were for males 40 differentially expressed genes (DEGs) and 73 DEGs for females with only 26 genes shared. The gene ontology (GO) term “response to bacterium” was shared, with several different DEGs contributing in males and females. Strongly upregulated genes TCN1 and CYP1B1 were unique to males and females, respectively. For male emphysema (E)-dominant and airway disease (A)-dominant COPD (defined by computed tomography) the term “response to stress” was found for both sub-phenotypes, but this included distinct up-regulated genes for the E-sub-phenotype (neutrophil-related CSF3R, CXCL1, MNDA) and for the A-sub-phenotype (macrophage-related KLF4, F3, CD36). In E-dominant disease, a cluster of mitochondria-encoded (MT) genes forms a signature, able to identify patients with emphysema features in a confirmation cohort. The MT-CO2 gene is upregulated transcriptionally in bronchial epithelial cells with the copy number essentially unchanged. Both MT-CO2 and the neutrophil chemoattractant CXCL1 are induced by reactive oxygen in bronchial epithelial cells. Of the female DEGs unique for E- and A-dominant COPD, 88% were detected in females only. In E-dominant disease we found a pronounced expression of mast cell-associated DEGs TPSB2, TPSAB1 and CPA3. The differential genes discovered in this study point towards involvement of different types of leukocytes in the E- and A-dominant COPD sub-phenotypes in males and females.

## Introduction

Chronic obstructive pulmonary disease (COPD) was third on the global mortality list of diseases in 2015^1^. COPD is caused by inhalation of noxious particles from cigarette smoke or open fires and this will trigger, in susceptible people, a chronic inflammatory process, which can severely damage the airways and the lung parenchyma^2^. Patients can present with both of these pathologies but some have predominant destruction of the parenchyma, termed emphysema, while others have predominant airway disease going along with thick airway walls. These features can be defined using computed tomography (CT) with image analysis to identify patients with emphysema-dominant (E-dominant) and airway disease-dominant (A-dominant) disease^3^.

Treatment of COPD patients with anti-inflammatory and broncho-dilator drugs can alleviate symptoms, but currently there is no cure for the disease. Therefore, understanding the inflammatory process and the genes involved in the process is crucial in order to develop new treatments. To this end, we recruited 534 patients and 280 controls in the Emphysema versus Airway Disease (EvA) study in 10 clinical centers in Europe^4^. Importantly patients recruited to this study were ex-smokers for at least one year and had no exacerbation of the disease in the preceding two months^4^ such that the inflammatory process in the lung is not biased by such external factors. To study the inflammatory process we obtained tissue samples from the airways and determined the transcriptome using RNA sequencing (RNAseq).

 Since there is accumulating evidence that gender has an impact on gene expression in various tissues and on inflammatory diseases^5–9^, we have hypothesized that the airway transcriptome will be distinct for male and female COPD. Furthermore, since it is emerging that COPD is not a single disease but consists of different entities, we have hypothesized that the transcriptome is distinct for males and females also in emphysema-dominant and airway disease-dominant sub-phenotypes of COPD.

Our analysis, in fact, demonstrates that there are many DEGs unique to males and females in the disease. And these DEGs point towards a role for neutrophils in male emphysema and for mast cells in female emphysema, while for male airway disease the DEGs support a role for macrophages.

## Results

### The transcriptome in COPD bronchial brush samples

Initially we explored the transcriptome of airway cells in COPD as such, without any separation of sub-phenotypes. RNA sequencing data for bronchial brush samples were obtained from 312 COPD cases and from 265 controls (see Table S1 for demographic details). Of the cases, a total of 259 cases was used as discovery set for identification of differentially expressed genes (DEGs), while the altogether 53 cases without CT data were not included and were used for confirmation (Table S2). Permutation analysis revealed 41 DEGs in COPD compared to controls (see Table S3, “male & female combined”). This included the top upregulated gene TCN1, a Vitamin B12 binding protein highly expressed in neutrophils and the top downregulated gene SCGB3A1, a gene expressed by club cells of the airway epithelium^10^. These findings suggest an increase of neutrophils and a decrease of club cells in the COPD lung.

Since mechanisms of inflammatory disease are known to be different for males and females^6–9^ we then analysed males (n = 209, 2/3 of samples) and females (n = 103, 1/3 of samples), separately.

Analysis of males alone revealed 40 DEGs including 9 DEGs not detected in the combined analysis (see Table S3, “male”). Of these, 20 were increased and 20 were decreased fold and the same pattern was confirmed for the confirmation group (Fig. S1A).

The dominant GO term associated with these 40 DEGs was “defense response” (LTF, IRAK3, PTGFR, SAA2, SCGB1A1, IL8, FOS, TPSAB1, SAA1, DPP4, GO:0006952, *p* = 0.00436). This included genes LTF, IRAK3, PTGFR, SCGB1A1, IL8, FOS that are also covered by the term “response to bacterium” (GO:0009617, *p* = 0.00924).

The top genes in our analysis (see Table S3) with a 2.5-fold higher expression in male cases were TCN1, highly expressed in neutrophils as noted above, CEACAM5, a cell adhesion molecule and CA12, a carbonic anhydrase, an enzyme involved in the conversion of CO_2_. A 2.5-fold lower expression was found for SCGB3A1.

In females using the same analysis strategy, we detected 73 DEGs (n = 86 female COPD versus n = 73 female controls, Table S3) including 40 DEGs not detected in the combined analysis (see Table S3, “female”). Only 26 DEGs were in common with males (marked in green in the Table S3). Among the 73 DEGs found in females 34 were increased and 39 were decreased in the discovery and the same pattern was found in the confirmation group (see Fig. S1B).

The dominant GO term associated with these 73 genes in females was “response to lipid” (LTF, TNC, LMO3, MAOB, IRAK3, WNT5B, PTGFR, ANXA3, SLIT2, SCGB1A1, AKR1C2, IL8, S100A14, GO: 0033993, *p* = 0.000618). A subset of these genes (LTF, MAOB, IRAK3, PTGFR, ANXA3, SCGB1A1, IL8, S100A14) was covered by the term “response to bacterium” (GO: 0009617, *p* = 0.0307). The “response to bacterium” genes LTF, IRAK3, PTGFR, SCGB1A1 and IL8 were shared with males while FOS in males and MAOB, ANXA3 and S100A14 in females were gender restricted.

For female COPD the top genes (see Table S3) with a 2.5-fold higher expression were CYP1B1, a cytochrome P450 member, which is involved in steroid metabolism, FGFBP1, a binding protein for fibroblast growth factor, which may enhance the activity of FGF, CEACAM5, a cell adhesion molecule, and CA12, a carbonic anhydrase involved in conversion of CO_2_. The expression of CA12 and CEACAM5 was also seen in the internal confirmation and it was confirmed by RT-PCR (see Fig. S2). Of note, the neutrophil associated gene TCN1, which we detected among all COPD cases and in the separate analysis of males, was not detected among females. A 2.5-fold lower expression in females was found for LTF, *i.e.* lactoferrin, an antimicrobial protein, and VGLL3, a co-factor for the TEA domain transcription factors. Also clearly decreased was SCGB3A1, a gene expressed by epithelial club cells^10^.

Taken together global analysis of gene expression in bronchial brush cells in COPD uncovers previously unknown differential expression for a multitude of genes. Our data show that a separate analysis of males and females uncovers many more differential genes (n = 87) compared to an analysis of males and females combined with only 41 DEGs. While males and females show a similar regulation of bronchial gene expression for genes like CA12 and CEACAM5, many of the DEGs are specific to either males or females. This includes TCN1 and SAA in males and CYP1B1 and VGLL3 in females. Also, the DEGs under the GO term “response to bacterium” showed a gender specific composition. This indicates that the pathophysiology of COPD is different for males and females. In order to further explore gender differences in COPD gene expression in the airways, we have looked into emphysema-dominant and airway disease dominant sub-phenotypes of the disease.

### Identification of differential genes for COPD sub-phenotypes in males

The EvA study has used CT image analysis to define emphysema-dominant (E) and airway disease-dominant (A) sub-phenotypes of COPD^3^. Using the RNAseq data on bronchial brush samples from these cases we analysed controls (n = 145 males) versus E-dominant (n = 50 males) and controls versus A-dominant cases (n = 32 males) and controls versus Eextreme (Eex, n = 22 males) and versus Aextreme (Aex, n = 16 males). Eex and Aex are the cases with the most pronounced CT-phenotype, which were selected by rank order of the CT measures of lung density and airway wall thickness^3^. The Eex and Aex samples are included in the E and A samples, respectively (see also under Supplement: Strategy for identification of DEGs for step by step explanation of data analysis).

With this approach we identified genes, which were unique to E-dominant or A-dominant disease with unique meaning that a given DEG was only significant for the controls versus E and not for the opposite controls versus A comparison—and vice versa.

For emphysema (E and Eex) we found at total of 51 unique DEGs. The eight top genes with a > 2.5-fold increase includes leukocyte associated genes like CSF3R (receptor for granulocyte colony stimulating factor) and ITGAX (a leukocyte integrin) and the HBB (hemoglobin-beta) gene (Table 1). Here expression of the CSF3R genes was increased more than threefold in Eex samples but there was only a minimal increase seen in Aex samples (Table 1, Table S4E).

**Table 1:** Top and informative differential genes unique to E and A sub-phenotypes of male COPD.

gene_name	ensembl_id	cpm_ctl	cpm_case	Ratio	FDR
**Top DEGs unique to the E sub-phenotype**					
ctl vs E					
HBB	ENSG00000244734.1	81.20	421.79	5.19	0.01959
SELL	ENSG00000188404.4	5.02	24.15	4.81	0.00001
IGHA1	ENSG00000211895.3	8.67	25.44	2.93	0.00773
S100A8	ENSG00000143546.5	20.73	56.03	2.70	0.00014
CSF3R	ENSG00000119535.13	7.74	24.45	3.16	0.02079
GPR97	ENSG00000182885.12	2.56	13.31	5.20	0.02805
ITGAX	ENSG00000140678.12	9.44	25.17	2.67	0.04854
HCAR3	ENSG00000255398.2	11.04	27.83	2.52	0.04379
CXCL1	ENSG00000163739.4	133.36	200.97	1.51	0.00038
MNDA	ENSG00000163563.7	13.53	29.91	2.21	0.04268
MT-ND1	ENSG00000198888.2	8087.88	13676.81	1.69	0.00836
MT-CO2	ENSG00000198712.1	9318.08	14155.29	1.52	0.04335
**Top DEGs unique to the A sub-phenotype**					
ctl vs A					
Metazoa_SRP	ENSG00000266422.1	20.22	71.16	3.52	0.00977
RN7SL1	ENSG00000258486.1	20.22	70.83	3.50	0.01000
RN7SL2	ENSG00000265150.1	34.67	115.33	3.33	0.01497
RNA45S5	ENSG00000225840.1	231.86	595.30	2.57	0.03197
KRT13	ENSG00000171401.10	9.90	47.72	4.82	0.0045
SPP1	ENSG00000118785.9	16.42	45.83	2.79	0,0300
TNC	ENSG00000041982.10	37.74	97.25	2,58	0.03118
CD36	ENSG00000135218.13	10.88	36.27	3.33	0.0467
F3	ENSG00000117525.9	199.98	324.69	1.62	0.01322
KLF4	ENSG00000136826.9	39.39	60.44	1.53	0.0452

The upper 8 DEGs in each sub-table show the highest differential expression, the remainder are informative genes analyzed in more detail in this study. Given are the unique DEGs in either E and A sub-phenotypes of COPD (see Table S4C and S5 for further detail). E = emphysema-dominant, A = airway disease-dominant, For resolution of E and A into extreme and median, upper and lower quartile see Table S4E.

HBB is thought to be an erythrocyte-restricted gene and its strong expression in airway brush samples comes unexpected. We therefore took a closer look and we confirmed in these samples the RNAseq data on increased expression in RT-PCR analysis (Fig. S4). Also, we could detect the transcripts in a lung cell line (data not shown) indicating that HBB likely can be expressed by bronchial epithelia.

For airway disease (A and Aex) there was a total of 89 unique DEGs. Among the eight top genes (> 2.5-fold above controls) there are SPP1 (osteopontin), KRT13 (keratin 13) and the macrophage associated CD36 receptor molecule. Of note, the expression of CD36 is increased more than threefold in Aex samples but there is no increase whatsoever in Eex samples (Table 1, Table S4E).

For the 51 DEGs unique to E and Eex the dominant GO terms is “response to stress” (Table S5), which covers 18 DEGs, many of which are among the top genes in Table 1. These 18 DEGs comprise a set of seven genes related to “mitochondrial ATP synthesis coupled electron transport” and a set of 10 genes that come under the term “defense response”. The latter set includes genes CSF3R, CXCL1 and MNDA, which are related to neutrophilic leukocytes.

Also for the 89 DEGs unique to A and Aex cases “regulation of response to stress” was the dominant GO term covering 14 DEGs. The “regulation of response to stress” also are related to leukocytes, but these genes, like CD36, F3 and KLF4, are associated with macrophages. These data suggest that there are distinct mechanisms of stress response operating in the E and A sub-phenotypes of COPD.

### String analysis in male COPD sub-phenotypes

In string analysis we noted for the significant A-DEGs there were 9 genes directly linked to the fos gene, including some of the “response to stress” genes (Figure S5B). DEGs in this cluster include the macrophage-associated genes CD36 and KLF4. This indicates that fos may have a central role in orchestrating inflammation in the bronchi of COPD patients with A-dominant disease.

**Figure 1:**
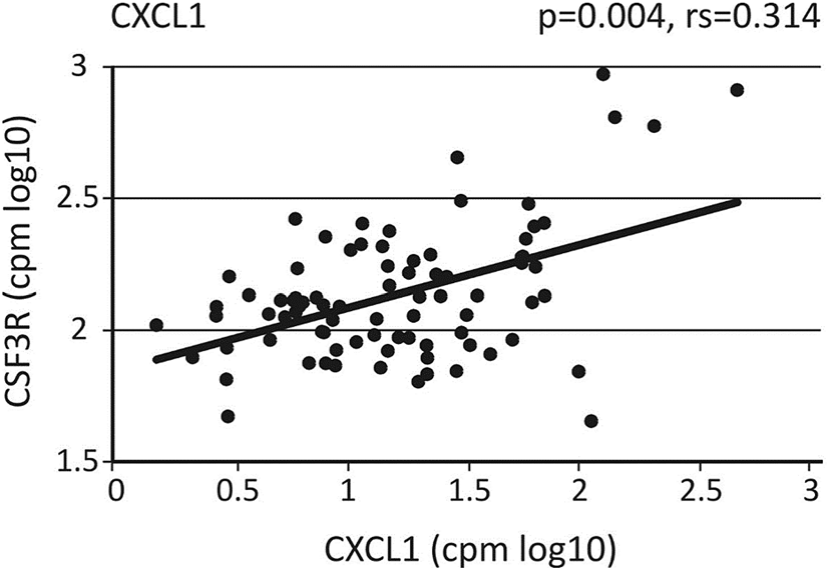
Association of the expression levels for neutrophil chemotactic CXCL1 with the neutrophil associated gene CSF3R. Shown are log transformed expression values in counts per million for CXCL1 and CSF3R in male A and E samples. *p*-values were determined using the Spearman’s rank correlation test.

For the E-DEGs there were interactions of the “response to stress” DEGs mentioned under GO terms and this includes the neutrophil associated genes MNDA, CSF3R and CXCL1 (Fig. S5A). The genes MNDA and CSF3R are expressed by neutrophils and their upregulation indicates the presence of neutrophils in the tissue. On the other hand, CXCL1 is a gene expressed by local tissue like bronchial epithelium and it is chemotactic for neutrophils. Transcript levels of CXCL1 in the bronchial brush samples showed a positive correlation with the CSF3R gene (Fig. 1). This suggests that CXCL1, expressed by bronchial epithelial cells, may be involved in recruiting neutrophils into the airways.

We then analysed the prominent string cluster formed by the eight mitochondrial DEGs (MT-ND1,2,3,5, MT-CO1,2,3, MT-ATP6, see Fig. S5A). These genes are increased in E-dominant COPD with a high expression level at around 10,000 cpm. The additional 5 of the total of 13 MT-encoded genes were also increased but are just below the cut-off. On the other hand, of the 80 nuclear-encoded genes only one showed a significant but minor increase (1.1-fold) at a very low expression level (Table S6). Hence, it appears that the increase of mitochondrial genes is specific for the MT-encoded genes while nucleus-encoded mitochondrial genes are not affected.

**Figure 2:**
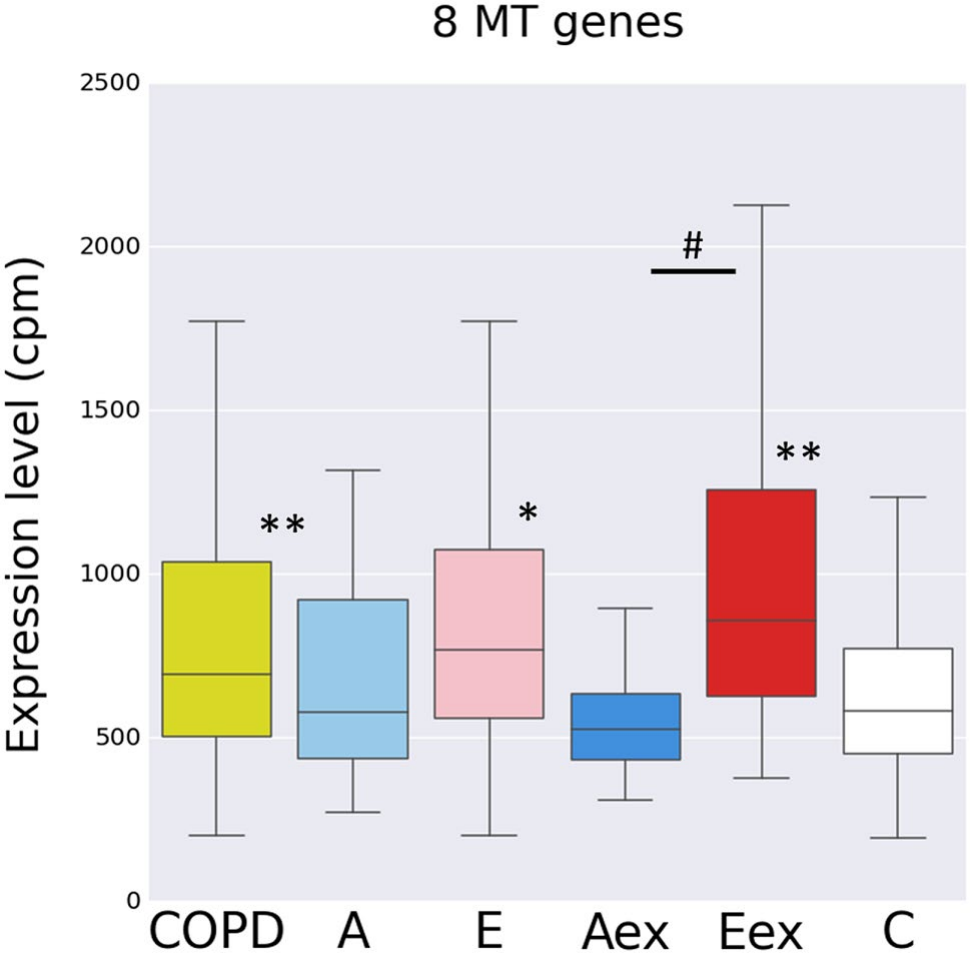
Gene expression level for eight genes encoded by the mitochondrium across all subgroups Shown is a box plot with median, upper and lower quartile and upper and lower whiskers for the sum of the expression level for the eight genes (MT-ND1,2,3,5, MT-CO1,2,3, MT-ATP6), Controls versus cases *p < 0.005; **p < 0.001; Eex versus Aex #*p* < 0.005, Wilcoxon rank sum test. COPD is all cases combined: E + A + mix + normal-CT. The figure was produced using the R package ggplot2 (Wickham H (2016). *ggplot2: Elegant Graphics for Data Analysis*. Springer-Verlag New York. ISBN 978-3-319-24277-4, https://ggplot2.tidyverse.org).

With the mitochondrial genes forming the most pronounced string cluster among E-DEGs we then looked at the RNAseq expression levels for the entire set of eight mitochondria-encoded genes (GO term: mitochondrial ATP synthesis coupled electron transport) in the COPD subgroups. Compared to controls the group of all COPD cases combined, the E and Eex cases but not the A and Aex cases showed significantly higher levels for these genes (Fig. 2).

Consistent with the association with the E-dominant COPD, the expression for these MT genes was related to severity of emphysema in that it showed a positive correlation with total lung capacity and a negative association with FEV1/FVC when tested across all COPD samples (FDR *p* < 0.05 for both using linear regression).

**Figure 3:**
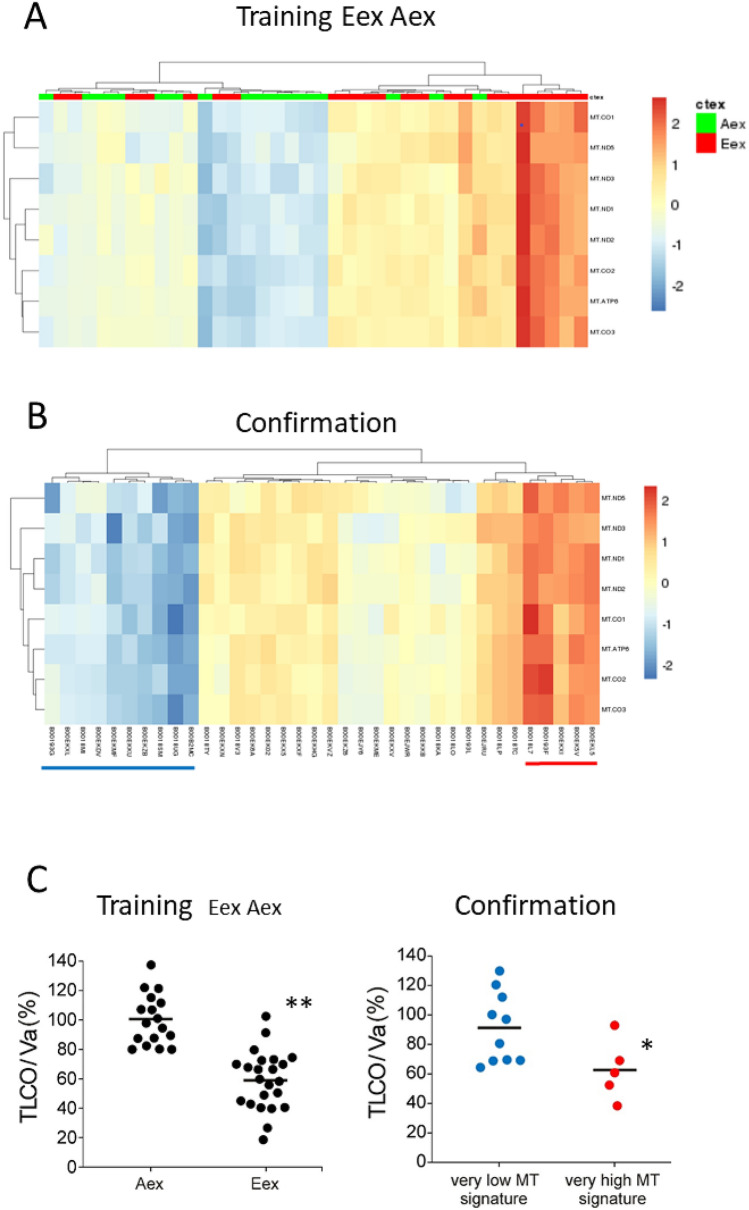
Mitochondrial signature for COPD sub-phenotypes The eight interacting mitochondrial genes were tested in un-supervised hierarchical clustering on Eex and Aex samples only (**A**) and on the confirmation samples (**B**). red = Eex; green = Aex. Fisher’s exact for 3A is 0.0036. (**C**) (left panel) gives TLCO/Va values for Eex and Aex samples (**p < 0.00001, Mann Whitney U test). (**C**) (right panel) gives the TLCO/Va values for the samples with very low MT gene expression marked in blue in Fig. 3B and for the samples with very high MT gene expression marked red in Fig. 3B (*p < 0.05, Mann–Whitney U test). **–-** = mean. The figure was produced using the R package pheatmap, pheatmap: Pretty heatmaps [Software] R Kolde, URL https://CRAN.R-project.org/package=pheatmap.

When using the eight MT genes in unsupervised hierarchical clustering we found a separation of Eex and Aex samples into a low level expression group dominated by Aex samples (green) and high level expression group dominated by Eex samples (red) (Fig. 3A).

In order to consolidate the MT signature we explored the 36 cases confirmation group, which had not been included in the original discovery group of samples. These cases were also separated into subgroups with high and low expression levels (Fig. 3B). Since for the confirmation group no CT data are available to determine the E or A phenotype we used the diffusion capacity TLCO/Va as a surrogate marker for CT-defined emphysema^3,11^. In our discovery group, TLCO/Va was significantly lower in E as compared to A patients (not shown) and in Eex as compared to Aex patients (see Fig. 3C). In the confirmation group, TLCO/Va levels were significantly lower in the 5 samples with very high MT gene levels as compared to the samples with very low expression levels (Fig. 3C). These data confirm that male patients with emphysema have higher expression levels of these MT genes. Taken together the MT signature can identify a sub-phenotype of COPD with characteristics of emphysema-dominant disease.

### Molecular analysis of MT-CO2 in COPD sub-phenotypes

A possible explanation for the increased transcript levels for the mitochondria-encoded genes in samples from the Eex patients is an increased number of mitochondria in the Eex brush material. To test this possibility we exploited the fact that mitochondria contain DNA, which codes for subunits of respiratory chain enzymes, including MT-CO2. Compared to genes in the nucleus the copy number for these genes per cell is much higher reflecting the number of mitochondria in a given cell.

**Figure 4:**
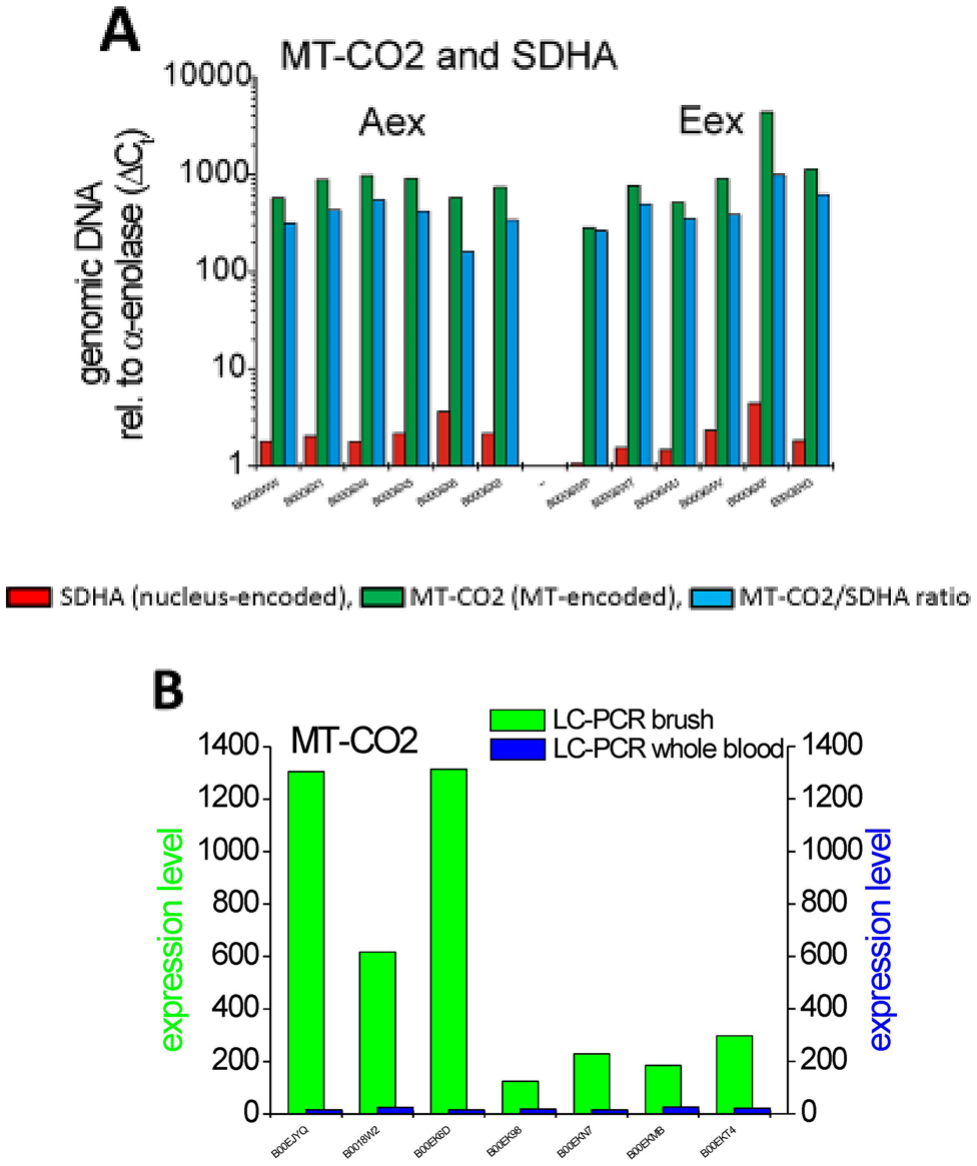
Molecular analysis of MT-CO2 in COPD sub-phenotypes. (**A**) Using genomic DNA for PCR, data of tested gene (MT-CO2 and SDHA) and housekeeping gene (alpha-enolase) were processed with the same settings (e.g. same thresholds). Cycle number of the housekeeping gene was subtracted from the corresponding gene and its absolute value was subsequently calculated to the power of 2. c_t_ = cycle number at threshold; MT-CO2/SDHA ratio Aex versus Eex *p* = 0.33 (Mann Whitney U-Test). The average level for MT-CO2 transcripts was 8255 ± 2938 cpm for Aex samples and 30,902 ± 10,200 cpm for Eex samples (**B**) MT-CO2 expression levels were determined by RT-PCR using primers given in Table S7 on brush samples and on whole blood samples from the same individuals. Expression levels were normalized to the levels of alpha-enolase. Samples 1–4 are Eex cases, samples 5–7 are Aex cases.

PCR on genomic DNA for the MT-CO2 gene in samples with low MT-CO2 transcripts (Aex) gave a 379-fold higher signal compared to the signal seen for the SDHA gene, which is encoded in the nucleus. When testing samples with high MT-CO2 transcripts (Eex), then the PCR on genomic DNA for MT-CO2 gave a 440-fold higher signal compared to the SDHA signal (Fig. 4A).

This value for MT-CO2 DNA is 1.2-fold higher in the Eex samples compared to the Aex samples, while the MT-CO2 mRNA expression is about 4.0-folder higher in these Eex samples compared to Aex samples with a mean 30,902 cpm and 8255 cpm, respectively. Hence, the higher MT-CO2 transcripts cannot be explained by a higher mitochondrial DNA copy number. These data indicate that the increased transcripts for mitochondrial genes in emphysema patient brush samples is not due to an increase in mitochondria, but reflect an increased prevalence of these transcripts in the presence of similar mitochondrial content of the cells.

Since the brush samples from patients with E-dominant disease also express several leukocyte-associated genes, one might speculate that leukocytes within the samples are responsible for the increased MT-transcript levels. To address this we determined the levels for MT-CO2 transcripts in whole blood samples from selected patients with high and low brush transcript levels. All blood samples contained very low transcript levels at around 20 relative units compared to the brush levels with 125 to 1305 relative units (see Fig. 4B), such that the blood levels are in average 25-fold lower compared to brush. This makes it unlikely that leukocyte derived MT-CO2 is responsible for the increased MT-CO2 levels in brush samples. It rather points towards an up-regulation of MT-CO2 transcripts in bronchial epithelium cells in E-dominant COPD.

### Interaction of MT genes and leukocyte associated genes

**Figure 5:**
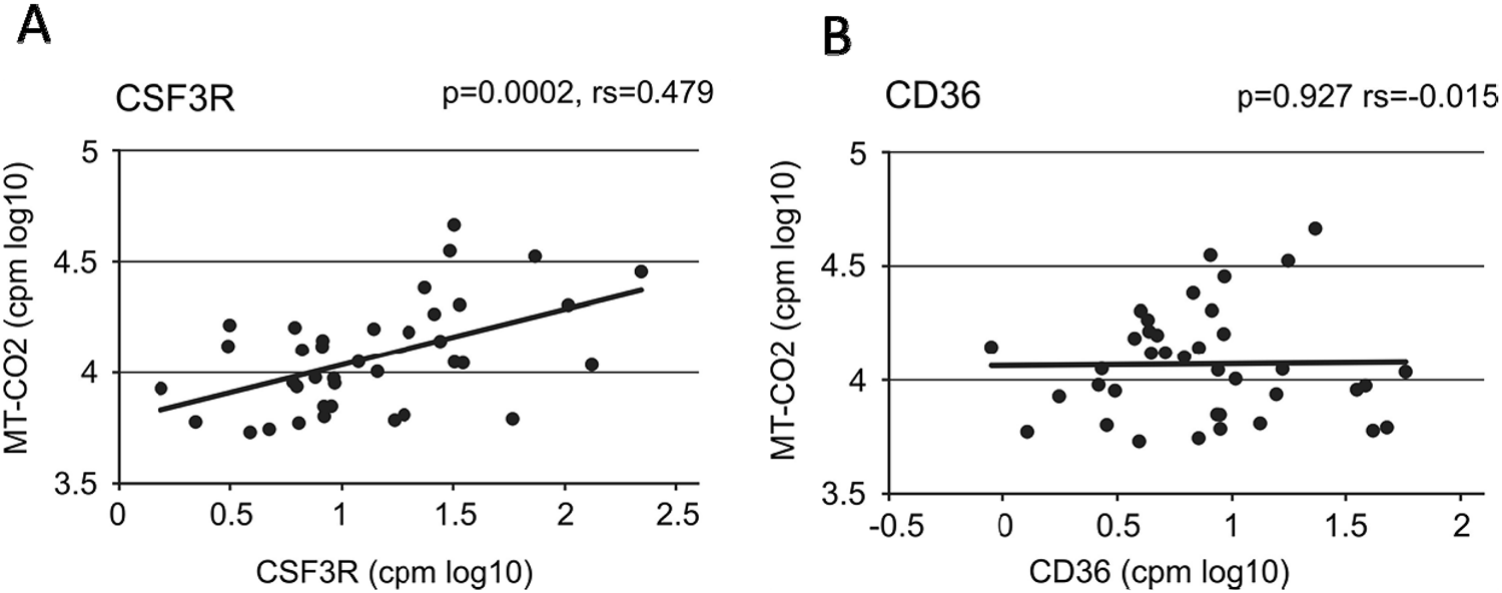
Association of MT-CO2 expression levels with neutrophil and macrophage marker genes. Shown are log transformed expression values in counts per million for MT-CO2 and CSF3R (**A**) and MT-CO2 and CD36 (**B**) in male Aex and Eex samples. *p*-values were determined using the Spearman’s rank correlation test. Analysis of A and E samples gave the same pattern with a significant correlation for CSF3R but not for CD36, *p* < 0.0003 and *p* = 0.062, respectively.

Analysis in the Eex and Aex samples of the expression levels demonstrates for MT-CO2 and the neutrophil specific CSF3R gene a significant association (Fig. 5A) while there is no association for MT-CO2 and the macrophage associated gene CD36 (Fig. 5B).

We then asked whether neutrophils might be able to induce transcription of mitochondria-encoded genes. However, co-culture of the human bronchial epithelial cell line 16 HBE and blood neutrophils with and without activation did not induce MT-CO2 in the bronchial epithelial cells (data not shown). We therefore turned to genes involved in neutrophil recruitment and looked at CXCL1, the transcripts of which are also selectively increased in emphysema dominant COPD (see Table S4C).

**Figure 6:**
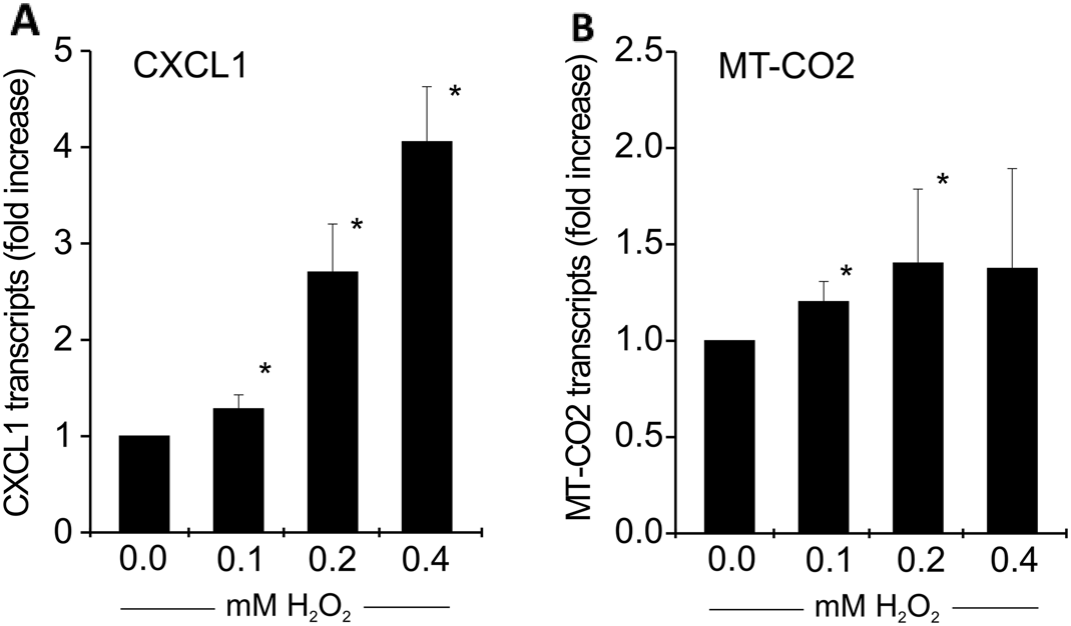
Induction of MT-CO2 and CXCL1 by oxygen peroxide. The effect of H2O2 on both CXCL1 and MT-CO2 transcripts was studied in the 16HBE cell line (human bronchial epithelial cells). Cells were treated with H2O2 for 4 hs, RNA was isolated and RT-PCR was performed (for method details see supplement). Results are normalized to alpha enolase and expressed as fold increase over untreated cells, n = 3 independent experiments, mean ± SD, *p < 0.05, Mann Whitney U Test. (a**)** CXCL1 (**b**) MT-CO2, *p < 0.05.

CXCL1 is a neutrophil chemoattractant and it is controlled by the transcription factor NF-kB^12^. Since reactive oxygen species (ROS) are known to be increased in COPD^13^ and since ROS can activate NF-kB^14^, we have tested the effect of H2O2 on the transcript level of CXCL1 in 16HBE. As shown in Fig. 6a, there is an up to fourfold increase of CXCL1 transcripts. At the same time the MT-CO2 transcripts are also 1.4-fold induced by H2O2 (Fig. 6b).

It remains to be determined whether the upregulation of MT-CO2 involves oxidative damage to mitochondrial DNA as has been shown in liver tissue^15^. In any event, the findings suggest that ROS may play a crucial sub-phenotype specific role in male patients with COPD in that it up-regulates both mitochondrial genes like MT-CO2 and neutrophil attracting chemokines like CXCL1.

### Identification of differential genes for COPD sub-phenotypes in females

Next, we analysed the female sample set, which includes half as many samples as the male sample set and therefore provides less statistical power. Still, when comparing gene expression in controls to the sub-phenotypes in females we found—when combining analysis at median, upper and lower quartile—a high number of DEGs. For controls versus E and Eex cases this was altogether 66 unique genes and for controls versus A and Aex cases we found 14 unique DEGs, with unique requiring an at least 1.5-fold difference and FDR < 0.05 only in comparison to one and not the other sub-phenotype (Table S4C). For the A and Aex DEGs in females there was no significant GO term. The dominant GO terms for the E + Eex DEGs were “regulation of apoptotic process” and “defence response”, which cover overlapping sets of genes, some of which are immune related (Table S5).

The patterns are clearly different from the results in males, for whom “response to stress” was found for the unique DEGs in both the E and A sub-phenotypes. String analysis for interactions revealed a group of 8 interacting DEGs for E-dominant cases (Fig S8) and neither this subset of DEGs nor the total set of sub-phenotype specific DEGs gave a significant separation in unsupervised hierarchical clustering (not shown).

### Top genes in female control versus E-dominant and control versus A-dominant comparisons

The analysis of control versus A-dominant female COPD revealed as top DEG (twofold higher compared to control) the BST2 (bone marrow stromal cell antigen 2, CD317) gene, which is expressed by various leukocytes, is induced by type I interferon and has antiviral activity^16^. Top genes expressed at > 2.5-fold higher levels in E-dominant brush samples included tryptase beta-2 (TPSB2), tryptase alpha-1, beta-1 (TPSAB1) and carboxypeptidase A3 (CPA3) the latter with a sixfold increase in gene expression over controls (see Table 2). All of the top genes are unique to females, except for SerpinB2, which is also increased in males, but here the gene was increased in A-dominant cases and not in E-dominant COPD.

**Table 2:** Top and informative differential genes unique to E and A sub-phenotypes of female COPD.

gene_name	ensembl_id	cpm_ctl	cpm_case	Ratio	FDR
**Top DEGs unique to the E sub-phenotype**					
ctl vs E					
FGFBP1	ENSG00000137440.3	11.55	44.12	3.82	1.71E−08
TPSB2	ENSG00000197253.8	19.49	58.64	3.01	0.04356
CDC20B	ENSG00000164287.8	26.30	68.74	2.61	0.00451
ANKRD18A	ENSG00000180071.12	11.17	28.90	2.59	0.02803
CPA3	ENSG00000163751.3	10.01	60.80	6.08	0.01515
TPSAB1	ENSG00000172236.11	32.77	115.12	3.51	0.00134
FKBP5	ENSG00000096060.9	45.85	149.93	3.27	4.32E−06
SERPINB2	ENSG00000197632.4	20.44	51,94	2.54	4.28E−06
**Top DEGs unique to the A sub-phenotype**					
ctl vs A					
BST2	ENSG00000130303.8	18.00	36.80	2.04	0.04016

Given are the unique DEGs in either E and A sub-phenotypes of COPD (see Table S4C and S5 for further detail). E = emphysema-dominant, A = airway disease-dominant, For resolution of E and A into extreme and median, upper and lower quartile see Table S4E.

Of note, the tryptases and carboxypeptidase A3 are mast cell associated genes^17^, suggesting a role for mast cells in the airways of emphysema-dominant COPD in females, but not in males.

The comparison of male and female unique DEGs for the COPD sub-phenotype reveals a limited overlap of 6 genes for E + Eex and 3 for A + Aex (Table S4C EA male, column CJ). Among the many gender exclusive genes were the 8 MT genes in males, none of which was shared with females.

On the other hand, there are altogether 71 DEGs only detected in female E + A brush samples (Fig. S9) and this includes genes like tryptase B2, tryptase AB1 and the peptidase CPA3.

Taken together when looking at COPD—irrespective of any sub-phenotype—26 DEGs are shared between males and females including CA12 and CEACAM5, but there are 14 DEGs only detected in male COPD and 47 DEGs only in female COPD.

The analysis of DEGs uniquely expressed in COPD sub-phenotypes also reveals clear gender differences. In males for the E-dominant sub-phenotype there is prominent expression of HBB and there is a mitochondrial gene signature. Also, in males there is a selective increase in E-dominant COPD of neutrophil associated genes CSF3R, MNDA and CXCL1. Here both CXCL1 and MT-CO2 are induced by H2O2 suggesting that in male emphysema reactive oxygen may provide an important pathophysiological mechanism. For females the top differential emphysema-restricted genes are the mast cell specific genes TPSB2, TPSAB1 and CPA3.

The cell type specific expression of these genes suggests a role for neutrophils in male E-dominant disease and a role for mast cells in female E-dominant disease.

The data indicate that COPD E-dominant and A-dominant sub-phenotypes are driven by different genes and different pathophysiological processes and that there is a strong gender effect.

## Discussion

In this study we were able to confirm our hypothesis that gender has an impact on gene expression in the airways of COPD and its sub-phenotypes. Importantly, we noted in male airway disease-dominant COPD an increased expression of macrophage associated genes, while in emphysema-dominant COPD neutrophil associated genes like CSF3R and CXCL1 were increased. At the same time E-dominant male COPD showed a pronounced transcriptional up-regulation of mitochondria-encoded genes like MT-CO2. The induction in a bronchial cell line of both of MT-CO2 and the neutrophil-attracting chemokine CXCL1 by oxygen peroxide suggests a role for reactive oxygen in the pathophysiology of male E-dominant COPD.

Looking at COPD as such, our study has uncovered a set of 40 differential genes in bronchial brush samples in male COPD as compared to control donors. Of these 20 DEGs were downregulated including secretoglobins SCGB3A1 and SCGB1A1. SCGB1A1 is strongly expressed by club cells and it has been reported to decrease with smoking^18^. Our finding of a decrease in ex-smoking COPD patients points toward a persistent damage to these cells in the disease. Kim et al*.*^19^ also focused on male patients and analysed gene expression in lung resection tissue from tumor patients with COPD. Their top 20 genes did not show any overlap with the DEGs in our study. This may be due to the fact, that the lung resection samples contain a mixture of many different cell types, while in our study the focus is on bronchial brush cells.

Morrow et al.^20^ also analyzed differential genes in resected lung tissue from COPD and controls. None of the 20 top DEGs in that study matched the DEGs we describe herein, which may be explained by the different sample types (whole lung tissue vs bronchial brush) and by the separate analysis of males and females in our study. In a transcriptome study on COPD bronchial brush samples Steiling et al.^21^ reported on 98 differential genes based on the analysis of 87 cases and 151 controls consisting of males and females and both current and ex-smokers. In the present study, we detected 60 genes not picked up in the study by Steiling et al.^21^. This indicates that although our criteria are more stringent we identify additional genes (see Venn diagram in Fig S3). The lower number of differential genes in our study may be due to our use of a 10 cpm differential cut-off and to a higher cut-off for fold-change (1.5-fold) compared to 1.25-fold in Steiling et al.^21^. Also, in our study current smokers and cases with recent exacerbations were excluded such that the focus is on the disease process itself. The detection of additional genes may be due to the sensitivity of RNA sequencing technology used and the difference in the composition of the patient population. In our study only individuals of European descent were included such that we have a homogeneous genetic background, which may allow for a better detection of DEGs.

While the top DEGs in our study were also detected in our internal confirmation group, an independent confirmation of these findings in a sufficiently powered airway transcriptome analysis is still required.

Analysis of differential genes unique to emphysema-dominant and to airway disease dominant COPD revealed response to stress genes in both sub-phenotypes. The “response to stress” DEGs unique for E-dominant COPD include genes associated with neutrophils like CSF3R, MNDA and CXCL1^22–24^ and this suggests that neutrophils might be selectively increased in the airway brush samples from emphysema-dominant disease. This assumption is in line with the recent demonstration that the 18FDG uptake in the upper zone of the lung is strongest in COPD patients with the lowest lung density as defined by CT densitometry^25^ and with the finding of increased blood neutrophils in E-dominant COPD^3^. For the A dominant phenotype CD36, F3 and KLF4 were found among the unique DEGs and these are associated with macrophages^26,27^. Increased numbers of macrophages and a shift to smaller macrophages in the COPD lung have been noted before^28^ but this has not been linked to CT-defined phenotypes in those studies. The associations of different leukocyte signatures in different sub-phenotypes as shown herein suggest that different mechanisms of inflammation operate in the airways of E-dominant and A-dominant COPD. The question whether the differential leukocyte signature can be used to predict diagnosis of COPD sub-phenotypes will require analysis of a larger group of defined COPD patients. Also, additional studies, including single cell sequencing, are required to confirm the presence of neutrophils in E-dominant and not in A-dominant disease and the presence of macrophages in A-dominant and not in E-dominant disease in male COPD patients.

Of note, the direct comparison of E-dominant cases and A-dominant cases did not give any significant DEGs in our analysis. Only when we added the higher statistical power of a large group of control donor samples and with that compared controls versus A-dominant cases and controls versus E-dominant cases then we found unique DEGs, which were specific to A-dominant disease and vice versa.

Another unique set of genes found upregulated in E-dominant male COPD are genes encoded in the mitochondrial genome and involved in oxidative phosphorylation, i.e. MT-CO1, MT-CO2, MT-CO3, MT-ND1, MT-ND2, MT-ND3, MT-ND5 and MT-ATP6. An increase of MT-CO1 and 12 s rRNA encoded in the mitochondrial genome has been reported in skeletal muscle tissue in COPD but nuclear encoded genes have not been studied and patients have not been dissected into sub-phenotypes^29^. An increased expression of MT-encoded genes has been noted recently in the alveolar space in COPD and this included MT-CO2 and MT-ND4^30^. Also, a selective increase in MT-encoded genes, has only been reported in liver tissue after oxidative damage of mitochondrial genes^15^. Our finding of induction of MT-CO2 by H_2_O_2_ in bronchial epithelial cells would be consistent with this mechanism.

One intriguing biological question is why the MT-encoded genes and the neutrophil-associated genes are co-expressed in E-dominant COPD. Here we focused on CXCL1, which can be expressed by various cells including neutrophils and epithelial cells^31,32^. The co-induction of both MT-CO2 and CXCL1 by H2O2 in a bronchial epithelial cell suggest that reactive oxygen species may be the crucial link between these genes in E-dominant COPD.

Analysis of DEGs in female COPD-sub-phenotypes revealed many genes with strong differential expression that do not show a differential expression in males at all or they show a reverse pattern. Also in normal-CT and mixed COPD, females express genes not seen in male samples (see supplement DEGs in mixed and mild sub-phenotypes of female COPD and Tables S4A and S4C). Such a differential gene expression is consistent with findings of gender specific immune responses, which may be due to effects by genes of the system expressed on sex chromosomes and to effects of sex hormones on the immune response^7^. When it comes to the innate immune system, female volunteers show a stronger rise in C-reactive protein and tumor necrosis factor in response to intravenous lipopolysaccharide^6^. Furthermore, when looking at the response to smoke inhalation different genes were induced in blood leukocytes in females and males^8^. Our data in females give support to the gender specific gene expression in COPD sub-phenotypes. A notable finding in female E-dominant COPD is the expression of mast cell associated genes like tryptase B2, tryptase AB1 and the peptidase CPA3, suggesting a role for mast cells in the pathophysiology of emphysema dominant COPD in females.

### Limitations

At this point there is no sizable data set on a bronchial brush transcriptome in COPD available, such that our findings cannot be analyzed in an external validation cohort. In the present study we have used an internal confirmation group, which was able to confirm several of the findings. This confirmation group has limitations, since it lacks the CT image data and there is no separate control group to go along with this patient group. RNA sequencing data have been confirmed for selected genes by RT-PCR, but these transcript data have not been confirmed at the protein level. Also, since we have analyzed material from a single bronchial brush in mild-to-moderated COPD it remains to be shown whether our findings also apply to severe COPD and whether it is a constant feature in any given patient.

 Taken together our data show unique sets of genes in COPD sub-phenotypes pointing towards a role of neutrophils in male E-dominant disease, a role for macrophages in male A-dominant disease and a role for mast cells in female E-dominant disease.

## Material and methods

(See supplement for methods of RNAseq, RT-PCR and cell culture).

Patients with mild to moderate COPD and controls were recruited after informed consent based on approval by the respective local Ethics Committees as part of the EvA study^4^.

Specifically, for the German centers a central approval was obtained from Ethic Committee of the University Hospital Munich (# 400-07), which covers the approvals from the Ethics Committee of Philipps-University of Marburg, the Albert-Ludwigs-University of Freiburg, the Medizinische Hochschule Hannover and the Ethic Committee of the University Hospital Munich. For Budapest, the study was approved by the Medical Research Council Budapest, Hungary (ETT-TUKEB, 22-278/2007-1018EKU). For Ferrara, the Ethics Committee of the Province of Ferrara, Italy approved (Nr. 071195 (2007). For Warszawa, it was the Ethics Committee of the National Tuberculosis and Lung Diseases Research Institute Warszawa, Poland (KE-51/2008). For the UK centers a central approval was obtained from NHS Research Ethics Committee of Leicestershire, Northamptonshire and Rutland Research Ethics Committee 2 (08/H0402/19). All methods were performed in accordance with the in accordance with relevant guidelines and regulations including the Declaration of Helsinki.

Chest CT scans were used to define sub-phenotypes based on lung density and airway wall thickness^3^. Flexible bronchoscopy with bronchial brushing and bronchoalveolar lavage was performed with mild sedation in supine position. Bronchial brush samples were taken from the right upper lobe S1, 2 and 3 segment bronchi and their sub-segments with a protected brush (5 mm diameter at bristle level, #BC-202D 5010; Olympus, Hamburg, Germany). The brush samples were transferred into RNAprotect immediately and stored at − 20 °C. The bronchoscopy procedure was done on altogether 699 EvA probands (419 cases, 280 controls) and went along with a low number of side effects in that post-bronchoscopy bronchitis was noted in two controls donors and seven patients and a pneumothorax in two patients. Pneumothorax required hospitalisation and patients recovered within a few days.

All samples were extracted using the AllPrep DNA/RNA Mini Kit (#80204, Qiagen, Hilden, Germany) on a Qiacube robot. After extraction, RNA concentrations were measured by UV quantification (NanoDrop 8000, in duplicate). RNA quality was tested by running the samples on a Bioanalyzer 2100 from Agilent, using the RNA6000 Nano Labchip kit (#5065-4476, Agilent Technologies, Inc., Santa Clara, CA), and allowed to calculate the RIN value. 390 samples were excluded from RNAseq early on because the RIN was < 5 or the total RNA amount < 1 µg.

The present study focusses on brush samples from patients with complete CT imaging data of the chest and patients were divided into subgroups based on CT phenotypes^4^. These were emphysema-dominant (E), airway disease-dominant (A), normal-CT and mixed phenotypes. E-cases had a low lung density (15^th^ percentile of lung density below − 925.6 HU) and little evidence of airway disease (% wall area in the right S1 bronchus < 69.3%). A-cases had little evidence of emphysema (15^th^ percentile of lung density above − 925.6 HU) and evidence of airway disease with % wall area the right S1 bronchus > 69.3%^3^. These cut-offs represent lower limit of normal and upper limit of normal for lung density and wall area, respectively, as determined in a control population of apparently healthy controls as described^3^.The most extreme 50% of the E and A groups were labelled Eex and Aex. For comparison, brush samples from healthy controls were processed alongside. For the present study only controls with FEV1/FVC of > 0.75 were used in order to exclude any possible borderline samples with COPD features. Cases without complete CT data were not used for the discovery but only for confirmation of DEGs (see Table S2 for breakdown of samples).

### Statistical and bioinformatics analysis

Raw read counts were normalized to ‘cpm’ (counts per million) with the TMM method of the edger R package^33^, which takes into account different library sizes and different RNA compositions across samples. Permutation tests were used to detect differential gene expression between groups of interest. Let Y be a random variable representing the absolute value of the difference in the median, upper or lower quartile of gene expression between the two groups. The exact distribution of Y, p(Y), under the null hypothesis that the distribution of gene expression in the two groups was the same was derived by considering all possible permutations of the group labels (case and control). The *p*-value of the observed absolute value of the difference x is then given as p(Y >  = x), the proportion of permutations where Y >  = x. It should be noted that this is essentially the same procedure as used for the Fisher’s exact test, and that this test can therefore be considered as an exact permutation test for quartile differences. The code for the permutation test is available at GitHub under https://github.com/MarcosFernandez/diffExprPermutation. The permutation approach has the advantage that it is very robust to outliers. Also, parametric approaches fail when the distribution assumptions are not met. Finally, the permutation technique can take into account the shape of the distribution such that upper and lower quartiles can be analyzed.

A gene was regarded as being significantly differentially expressed if the p-value from the permutation test (corrected for multiple testing) was < 0.05, the absolute difference in median, upper or lower quartile expression was ≥ 10 cpm and the absolute fold change in expression was ≥ 1.5. Gene ontology enrichment analysis was performed with DAVID 6.8^34^ and Gprofiler^35^ and gene interactive networks were constructed with STRING v10.5^36^. Heatmaps were drawn with the ‘pheatmap’ R package (https://www.R-project.org/) scaling by row. Group comparisons were done with Mann Whitney U-Test. Comparison of RNAseq and RT-PCR for gene expression analysis was performed using Spearmann’s R test (www.sofastatistics.com), Version 1.4.3).

## Supplementary Information

Supplementary Information.

Supplementary Table S4A.

Supplementary Table S4B.

Supplementary Table S4C.

Supplementary Table S4D.

Supplementary Table S5.

Supplementary Table S6.

## Data Availability

Data: The accession code for the bronchial brush transcriptome data is EGAC00001000389. Applicants will be authorized by the data accession committee after having submitted a project describing the intended analysis. Biological material: The study uses bronchial brush material from cases and controls. There is limited material remaining that can be released upon request.

## Abbreviations

A-dominant COPD: Airway disease-dominant COPD = COPD with increased airway wall thickness
Aex: Aextreme = airway disease-dominant COPD with most pronounced CT phenotype
COPD: Chronic obstructive pulmonary disease
cpm: Counts per million
CT: Computed tomography
DEG: Differentially expressed gene
E-dominant COPD: Emphysema-dominant COPD = COPD with decreased lung density
Eex: Eextreme = emphysema-dominant COPD with most pronounced CT phenotype
EvA: Emphysema versus airway disease project
FEV1: Forced expiratory volume in 1 s
FVC: Forced vital capacity
LASSO: Least absolute shrinkage and selection operator
mixed COPD: COPD with both low lung density and high airway wall thickness
MT: Mitochondria
normal CT COPD: COPD with lung density and airway wall thickness in the range of apparently healthy controls
RNAseq: RNA sequencing
TLCO/Va: Transfer factor of the lung for carbon monoxide/alveolar volume
