# Supplementary material for: Gender specific airway gene expression in COPD sub-phenotypes supports a role of mitochondria and of different types of leukocytes
Source: Sci Rep. 2021 Jun 18;11:12848. doi: 10.1038/s41598-021-91742-x (PMC8213687; doi:10.1038/s41598-021-91742-x)
Supplement: Supplementary file 1 — Supplementary Information. [file 41598_2021_91742_MOESM1_ESM.pdf]

Supplement to

Gender specific airway gene expression in COPD sub-phenotypes supports a role of mitochondria and of different types of leukocytes

Anna Esteve-Codina[1], Thomas P. Hofer[2,21], Dorothe Burggraf[2], Marion S. Heiss-Neumann[2], Wolfgang Gesierich[2], Anne Boland[3], Robert Olaso[3], Marie-Therese Bihoreau[3], Jean-Francois Deleuze[3], Winfried Moeller[4], Otmar Schmid[4], María Soler Artigas[1], Kathrin Renner[5], Jens M. Hohlfeld[6], Tobias Welte[7], Thomas Fuehner[7], Lukas Jerrentrup[8], Andreas Rembert Koczulla[8], Timm Greulich[8], Antje Prasse[7], Joachim Müller-Quernheim[9], Sumit Gupta[10], Christopher Brightling[10], Deepak R. Subramanian[11], David G Parr[11], Umme Kolsum[12], Vandana Gupta[12], Imre Barta[13], Balázs Döme[14], János Strausz[15], Mariarita Stendardo[16], Marco Piattella[16], Piera Boschetto[16], Damian Korzybski[17], Dorota Gorecka[17], Adam Nowinski[17], Marc Dabad[1], Marcos Fernández-Callejo[1], David Endesfelder[18], Wolfgang zu Castell[18], Pieter S Hiemstra[19], Per Venge[20], Elfriede Noessner[21], Thasso Griebel[1], Simon Heath[1], Dave Singh[12], Ivo Gut[1], Loems Ziegler-Heitbrock[2,*]

Tables S4 A-D, S5, S6 are submitted as separate files

Contents Tables and Figures

Tables S1 Demographics for patients and controls
Tables S2 number of samples in CT phenotype groups
Tables S3A Differential genes in bronchial brush samples for male COPD cases versus controls
Tables S3B Differential genes in bronchial brush samples for female COPD cases versus controls
Tables S3C Comparison of the current combined male female analysis with results by Steiling et al.
Tables S4A to D: DEGs unique to E and A,
    see separate excel attachments
Table S4E Top differential genes unique to E and A sub-phenotypes of male and female COPD
Table S5 Gprofiler biological process enrichment for cases and E A sub-phenotypes,
    see separate excel attachment
Table S6 all mitochondrial genes male Eex,
    see separate excel attachment
Tables S7 PCR primers and products
Tables S8 xCELL deconvolution analysis of EvA transcriptome E vs A

Figure S1A: semi-supervised hierarchical clustering of the male COPD DEGs
Figure S1B: semi-supervised hierarchical clustering of the female COPD DEGs
Figure S1C: 3D plot of top 3 LASSO DEGs in males
Figure S1D: 3D plot of top 3 LASSO DEGs in females
Figure S 2 CEACAM5 and CA12 expression and confirmation of RNAseq expression levels by RT-PCR.
Figure S3 Venn diagram for bronchial brush genes compared to the 98 genes of Steiling *et al.*
Figure S 4 Confirmation of HBB RNAseq expression levels by RT-PCR
Figure S5 Interaction analysis for genes upregulated in E and A sub-phenotypes of male COPD.
Figure S6 Confirmation of RNAseq expression levels by RT-PCR
Figure S7 Un-supervised hierarchical clustering of MT-genes, macrophage associated genes and neutrophil associated genes
Figure S8 Interaction analysis for genes upregulated in E and A sub-phenotypes of female COPD
Figure S9 Venn diagram of male versus female E and A associated genes

Table S1 Demographics for patients and controls

			case male (n=209)			case female (n=103)		male/female comparison
			median	IQR		median	IQR	
age	years		66	9		63	9	p<0.01*
smoking history	packyears		38,75	28,75		33,00	19,25	p<0.01
FEV1	% predicted		0,72	0,27		0,76	0,24	p<0.01
FEV1/ FVC	%		58,16	15,53		60,94	11,62	p<0.05
			E-dominant male (n=50)			E-dominant case female (n=24)		
			median	IQR		median	IQR	
age	years		67,5	8		64	11,75	ns
smoking history	packyears		36,75	32,63		39,5	19,5	ns
FEV1	% predicted		0,65	0,23		0,68	0,19	ns
FEV1/ FVC	%		51,56	15,17		54,5	12,62	ns
			A-dominant male (n=32)			A-dominant case female (n=17)		
			median	IQR		median	IQR	
age	years		66	7,5		61	7	p<0.05
smoking history	packyears		39,75	29,81		27,5	15,5	p<0.05
FEV1	% predicted		0,67	0,25		0,80	0,22	p<0.05
FEV1/ FVC	%		56,53	16,06		64,82	11,25	p<0.05
			confirmation cases (n= 36)			confirmation cases (n= 17)		
			median	IQR		median	IQR	
age	years		65	10,5		63	10	ns
smoking history	packyears		31,25	19,00		35	21,35	ns
FEV1	% predicted		0,72	0,23		0,76	0,18	p<0.05
FEV1/ FVC	%		58,50	15,62		60,7	10,35	ns
			control male (n=168)			control female (n= 97)		
			median	IQR		median	IQR	
age	years		60	15		56	14	p<0.05
smoking history	packyears		22,15	24,5		10	23,75	p< 0.001
FEV1	% predicted		1,07	0,20		1,15	0,23	p<0.05
FEV1/ FVC	%		80,23	4,78		82	5,2	p<0.01

all parameters in cases are p<0.005 compared to controls

* for all comparisons Student's T-test was used, ns= not significant

Table S2 number of samples in CT phenotype groups

	male brush samples				female brush samples			Total
**case**	**209**				**103**			312
emphysema	50	Eex	22		24	Eex	16	
		non Ex	28			non Ex	8	
airway disease	32	Aex	16		17	Aex	4	
		non Ex	16			non Ex	13	
mixed	10	MixEx	6		7	MixEx	3	
		non Ex	4			non Ex	4	
mild	81	MildEx	46		38	MildEx	19	
		non Ex	35			non Ex	20	
Total	173				86			259
noCT validation	36				17			53
**control**	**168**				**97**			265
controls >0.75	145				73			218
controls <0.75	23				24			47

Cases were classified as emphysema dominant, airway disease dominant, mixed and mild based on quantitative CT analysis, and each group was subdivided in those without or with the most extreme features of the CT measures (see Subramanian et al for details).

The noCT validation samples were not used for discovery of DEGs but only for validation

controls > 0.75 are controls who have an FEV1/FVC ratio of greater than 0.75 such that they lung function is clearly separated from the COPD specific cut-off level of 0.70. Only these controls were used.

Table S3A Differential genes in bronchial brush samples for male COPD cases versus controls

See also separate excel attachment

Table S3 ctl vs cases DE genes male female						
**A**		**male**				
CTL vs COPD at median 1.5x p<0.05 male						LASSO
gene_name	ensembl_ID	cpm ctl	cpm cases	ratio	FDR	uncorrelated predictors
TCN1	ENSG00000134827.3	4,93	17,80	3,61	1,48E-11	
CA12	ENSG00000074410.9	9,72	27,03	2,78	3,83E-14	99
CEACAM5	ENSG00000105388.10	83,93	227,34	2,71	2,54E-13	1
RN7SL2	ENSG00000265150.1	32,78	82,76	2,52	1,80E-02	4
Metazoa_SRP	ENSG00000266422.1	19,43	48,30	2,49	6,35E-03	
RN7SL1	ENSG00000258486.1	19,40	47,89	2,47	6,98E-03	
FGFBP1	ENSG00000137440.3	9,92	23,76	2,39	1,59E-10	
SAA1	ENSG00000173432.6	96,81	182,20	1,88	6,19E-07	5
SAA2	ENSG00000134339.4	45,66	84,09	1,84	1,71E-05	2
NEAT1	ENSG00000245532.3	169,58	307,91	1,82	2,69E-03	5
RNA45S5	ENSG00000225840.1	224,04	404,37	1,80	3,03E-02	4
MUC13	ENSG00000173702.3	61,88	109,90	1,78	3,26E-07	1
IL8	ENSG00000169429.6	38,75	68,52	1,77	8,90E-05	3
TNC	ENSG00000041982.10	37,02	64,57	1,74	6,55E-06	7
PSCA	ENSG00000167653.4	209,68	353,87	1,69	2,48E-05	48
UPK1B	ENSG00000114638.3	81,87	132,79	1,62	3,99E-06	1
RP11-124N14.4	ENSG00000229124.1	19,04	30,47	1,60	1,50E-13	56
IRAK3	ENSG00000090376.4	22,73	35,25	1,55	6,71E-12	13
SLC5A8	ENSG00000256870.2	32,77	49,98	1,53	7,33E-08	2
FOS	ENSG00000170345.4	20,98	31,92	1,52	6,11E-03	3
LPPR3	ENSG00000129951.13	33,04	21,86	0,66	3,06E-04	1
FOLR1	ENSG00000110195.7	81,97	53,63	0,65	1,59E-09	1
LMO3	ENSG00000048540.10	56,06	36,54	0,65	4,94E-09	5
C16orf89	ENSG00000153446.11	115,94	75,05	0,65	1,03E-10	34
NTS	ENSG00000133636.5	137,12	86,71	0,63	1,72E-04	35
PTGFR	ENSG00000122420.5	85,78	53,95	0,63	5,29E-15	52
TNS3	ENSG00000136205.12	59,12	36,98	0,63	7,84E-15	7
CCDC81	ENSG00000149201.5	72,59	43,00	0,59	1,63E-13	93
FHOD3	ENSG00000134775.10	28,65	16,83	0,59	6,33E-11	20
CLDN8	ENSG00000156284.4	40,31	23,41	0,58	7,89E-12	36
TPSAB1	ENSG00000172236.11	27,34	15,09	0,55	1,97E-02	3
DPP4	ENSG00000197635.5	22,45	12,38	0,55	3,03E-12	58
LTF	ENSG00000012223.7	113,45	56,69	0,50	1,23E-07	2
GPR116	ENSG00000069122.13	22,09	10,98	0,50	9,12E-12	4
CYP2A13	ENSG00000197838.4	41,03	20,14	0,49	5,29E-15	54
SCGB1A1	ENSG00000149021.2	27289,36	13331,51	0,49	8,24E-13	90
SEC14L3	ENSG00000100012.7	92,97	44,40	0,48	1,13E-10	3
HSD17B13	ENSG00000170509.7	137,44	61,36	0,45	2,90E-12	22
SCGB3A1	ENSG00000161055.3	618,43	241,06	0,39	6,82E-06	3
CTD-2531D15.4	ENSG00000255446.1	660,93	248,04	0,38	2,17E-10	44

Green tag: genes shared with the opposite sex, yellow tag: top uncorrelated predictors in LASSO

Table S3B Differential genes in bronchial brush samples for female COPD cases versus controls
See also separate excel attachment

Table S3 ctl vs cases DE genes male female						
**B**		**female**				
CTL vs COPD at median 1.5x p<0.05 female						LASSO
gene_name	ensembl_ID	cpm ctl	cpm cases	ratio	FDR	uncorrelated predictors
CEACAM5	ENSG00000105388.10	36,04	186,37	5,17	9,73E-11	1
CYP1B1	ENSG00000138061.7	3,89	18,63	4,78	6,02E-08	7
FGFBP1	ENSG00000137440.3	7,51	26,71	3,56	3,17E-11	27
CA12	ENSG00000074410.9	7,01	21,17	3,02	4,12E-09	41
RN7SL1	ENSG00000258486.1	13,36	35,41	2,65	2,27E-02	3
Metazoa_SRP	ENSG00000266422.1	13,40	35,48	2,65	2,29E-02	3
CPA3	ENSG00000163751.3	9,82	21,48	2,19	1,06E-02	3
DPYSL3	ENSG00000113657.8	34,75	71,64	2,06	2,01E-06	8
UPK1B	ENSG00000114638.3	60,23	119,75	1,99	5,55E-06	8
MUC5AC	ENSG00000215182.6	744,74	1400,50	1,88	4,57E-04	5
DSC3	ENSG00000134762.12	12,75	23,59	1,85	1,21E-07	54
SLC5A8	ENSG00000256870.2	28,29	52,03	1,84	1,10E-06	
FUT3	ENSG00000171124.8	20,67	37,53	1,82	2,80E-10	5
TFCP2L1	ENSG00000115112.7	22,85	40,88	1,79	2,77E-06	4
TNC	ENSG00000041982.10	29,47	52,50	1,78	1,32E-06	48
NEAT1	ENSG00000245532.3	159,75	278,60	1,74	3,30E-02	15
IL8	ENSG00000169429.6	34,05	59,10	1,74	3,42E-03	11
AKR1C2	ENSG00000151632.11	62,16	106,13	1,71	1,68E-05	8
PARM1	ENSG00000169116.7	23,18	38,91	1,68	1,11E-07	35
SERPINB5	ENSG00000206075.9	23,78	39,48	1,66	1,88E-06	
J01415.23	ENSG00000211459.2	2002,38	3295,73	1,65	3,84E-04	
ANXA3	ENSG00000138772.8	21,77	35,83	1,65	8,74E-09	35
IRAK3	ENSG00000090376.4	20,20	32,96	1,63	9,03E-11	12
MT-ND1	ENSG00000198888.2	7022,69	11327,10	1,61	2,88E-04	3
MSLN	ENSG00000102854.9	53,23	85,29	1,60	1,41E-03	23
MIR4485	ENSG00000210082.2	9761,56	15512,12	1,59	2,01E-04	1
MALAT1	ENSG00000251562.3	220,30	348,41	1,58	3,41E-03	5
CD44	ENSG00000026508.11	95,14	150,11	1,58	2,73E-04	
S100A14	ENSG00000189334.4	69,53	109,46	1,57	4,80E-07	1
SLC26A2	ENSG00000155850.7	41,84	65,13	1,56	5,95E-06	
MT-CO3	ENSG00000198938.2	9961,92	15463,21	1,55	2,02E-03	3
MUC13	ENSG00000173702.3	63,96	98,82	1,55	1,15E-02	
NEK6	ENSG00000119408.12	21,19	32,29	1,52	7,15E-05	18
CCNL1	ENSG00000163660.7	19,29	29,38	1,52	4,72E-03	
SCD	ENSG00000099194.4	59,23	39,48	0,67	3,74E-02	28
SSBP4	ENSG00000130511.9	35,27	23,51	0,67	1,97E-02	1
MT2A	ENSG00000125148.6	62,60	41,72	0,67	7,33E-03	
C2orf40	ENSG00000119147.5	222,97	148,54	0,67	7,77E-05	25
MAOB	ENSG00000069535.11	74,26	49,43	0,67	4,50E-08	26
UNC93B1	ENSG00000110057.3	128,36	85,27	0,66	7,49E-04	
C14orf132	ENSG00000227051.4	55,91	36,97	0,66	2,87E-07	18
LMO3	ENSG00000048540.10	71,72	47,25	0,66	1,27E-05	2
PDLIM4	ENSG00000131435.8	45,63	29,70	0,65	7,33E-03	
LRP5	ENSG00000162337.7	46,94	30,24	0,64	8,25E-04	4
GAS6	ENSG00000183087.9	34,07	21,47	0,63	2,69E-04	
CCDC8	ENSG00000169515.5	29,97	18,71	0,62	1,27E-09	44
CAPS	ENSG00000105519.7	1602,64	994,86	0,62	5,09E-03	
TNS3	ENSG00000136205.12	65,96	40,65	0,62	4,51E-09	10
PTGFR	ENSG00000122420.5	97,86	59,80	0,61	3,76E-07	19
VMO1	ENSG00000182853.7	56,24	34,26	0,61	3,32E-03	1
ITM2A	ENSG00000078596.6	29,55	17,90	0,61	5,45E-05	4
CLDN8	ENSG00000156284.4	43,96	26,39	0,60	6,95E-07	20
DEGS2	ENSG00000168350.6	94,67	56,60	0,60	1,57E-03	
PROS1	ENSG00000184500.10	494,16	290,24	0,59	3,97E-08	4
LYPD2	ENSG00000197353.3	26,39	15,34	0,58	5,40E-03	10
SCGB1A1	ENSG00000149021.2	26961,11	15445,95	0,57	1,57E-05	6
AC104532.2	ENSG00000267314.1	26,27	14,86	0,57	2,00E-03	
WNT5B	ENSG00000111186.8	27,89	15,64	0,56	3,50E-11	13
SLIT2	ENSG00000145147.14	27,31	14,78	0,54	6,32E-07	23
GPR116	ENSG00000069122.13	29,40	15,78	0,54	1,80E-04	3
SERPINB7	ENSG00000166396.8	28,13	14,73	0,52	8,88E-07	86
CLDN3	ENSG00000165215.5	32,39	16,95	0,52	4,73E-03	2
METRN	ENSG00000103260.3	25,45	13,07	0,51	6,05E-04	
LPPR3	ENSG00000129951.13	39,82	20,08	0,50	1,12E-04	4
FHOD3	ENSG00000134775.10	34,67	16,97	0,49	1,24E-08	20
CYP2A13	ENSG00000197838.4	45,58	20,62	0,45	3,91E-09	65
SEC14L3	ENSG00000100012.7	104,63	46,81	0,45	2,73E-06	27
KCNA1	ENSG00000111262.4	18,39	8,16	0,44	4,98E-10	43
LTF	ENSG00000012223.7	150,03	61,79	0,41	1,07E-06	6
HSD17B13	ENSG00000170509.7	143,97	58,98	0,41	7,33E-09	58
VGLL3	ENSG00000206538.3	16,30	6,26	0,38	9,02E-09	75
CTD-2531D15.4	ENSG00000255446.1	691,68	263,66	0,38	3,10E-04	3
SCGB3A1	ENSG00000161055.3	924,61	327,86	0,35	3,20E-04	1

Green tag: genes shared with the opposite sex, yellow tag: top uncorrelated predictors in LASSO

Table S3C Comparison of the current combined male female analysis with results by Steiling et al. See also separate excel attachment

Table S3 ctl vs cases DE genes male female						
C	male & female combined					Genes also found in Steiling et al AJRCCM,187: 933, 2013
CTL vs COPD at median 1.5x p<0.05 male female						
gene_name	ensembl_ID	cpm ctl	cpm cases	ratio	FDR	
TCN1	ENSG00000134827.3	4,24	14,92	3,52	1,95E-16	yes
CEACAM5	ENSG00000105388.10	73,93	217,08	2,94	9,12E-23	yes
CA12	ENSG00000074410.9	8,65	24,30	2,81	1,73E-25	
CYP1B1	ENSG00000138061.7	6,48	17,35	2,68	2,12E-18	
FGFBP1	ENSG00000137440.3	9,12	23,87	2,62	3,97E-17	yes
RN7SL1	ENSG00000258486.1	17,32	40,74	2,35	5,97E-04	
Metazoa_SRP	ENSG00000266422.1	17,43	40,86	2,34	6,00E-04	
RN7SL2	ENSG00000265150.1	29,44	64,62	2,20	4,16E-03	
HBB	ENSG00000244734.1	89,93	188,78	2,10	3,20E-02	yes
UPK1B	ENSG00000114638.3	71,58	128,86	1,80	1,55E-11	yes
SAA1	ENSG00000173432.6	98,35	175,07	1,78	5,20E-08	yes
TNC	ENSG00000041982.10	33,29	57,82	1,74	1,18E-11	yes
MUC13	ENSG00000173702.3	63,59	107,78	1,69	2,73E-09	yes
NEAT1	ENSG00000245532.3	166,52	277,66	1,67	4,31E-04	
DPYSL3	ENSG00000113657.8	41,61	68,95	1,66	2,40E-15	yes
IL8	ENSG00000169429.6	38,62	63,85	1,65	1,49E-06	
SAA2	ENSG00000134339.4	47,99	78,60	1,64	2,61E-05	yes
SLC5A8	ENSG00000256870.2	31,70	51,01	1,61	1,05E-12	yes
SERPINB5	ENSG00000206075.9	24,71	39,33	1,59	1,36E-13	
IRAK3	ENSG00000090376.4	21,60	34,09	1,58	1,29E-22	yes
FUT3	ENSG00000171124.8	25,40	39,61	1,56	6,82E-19	yes
RP11-124N14.4	ENSG00000229124.1	18,78	29,08	1,55	7,63E-16	
PSCA	ENSG00000167653.4	197,21	304,45	1,54	3,01E-05	
PARM1	ENSG00000169116.7	26,68	40,54	1,52	1,54E-21	
PROS1	ENSG00000184500.10	465,33	296,67	0,64	5,81E-16	
PTGFR	ENSG00000122420.5	87,85	55,72	0,63	1,54E-21	yes
CCDC8	ENSG00000169515.5	27,05	16,95	0,63	2,70E-19	
TNS3	ENSG00000136205.12	61,05	38,24	0,63	1,76E-22	yes
CCDC81	ENSG00000149201.5	76,68	46,46	0,61	8,55E-23	yes
LPPR3	ENSG00000129951.13	34,82	20,68	0,59	1,33E-07	
CLDN8	ENSG00000156284.4	42,01	23,88	0,57	6,24E-20	yes
SLIT2	ENSG00000145147.14	24,97	13,62	0,55	1,07E-18	yes
FHOD3	ENSG00000134775.10	30,77	16,76	0,54	1,02E-19	yes
SCGB1A1	ENSG00000149021.2	27200,17	14417,06	0,53	9,89E-18	
GPR116	ENSG00000069122.13	24,50	12,03	0,49	7,61E-14	yes
SEC14L3	ENSG00000100012.7	97,98	46,03	0,47	3,11E-17	
CYP2A13	ENSG00000197838.4	43,08	19,96	0,46	4,17E-26	
LTF	ENSG00000012223.7	122,31	55,13	0,45	5,17E-13	yes
HSD17B13	ENSG00000170509.7	141,20	61,18	0,43	1,29E-21	yes
CTD-2531D15.4	ENSG00000255446.1	688,34	248,93	0,36	2,60E-15	
SCGB3A1	ENSG00000161055.3	770,67	256,00	0,33	2,29E-10	

Tables S4 A to D DEG unique to E and A, see separate excel attachment

Table S4E male

Table S4E Top differential genes unique to E and A sub-phenotypes of male COPD

ctl vs E median						ctl vs A median			
**gene_name**	**ensembl_id**	**cpm_ctl**	**cpm_case**	**ratio**	**FDR**	**cpm_ctl**	**cpm_case**	**ratio**	**FDR**
HBB	G0000024473	81,20	421,79	5,19	0,01959	83,10	185,20	2,23	0,282

ctl vs E upper						ctl vs A upper			
**gene_name**	**ensembl_id**	**cpm_ctl**	**cpm_case**	**ratio**	**FDR**	**cpm_ctl**	**cpm_case**	**ratio**	**FDR**
SELL	G0000018840	5,02	24,15	4,81	0,00001	5,10	11,59	2,27	0,212
IGHA1	G0000021189	8,67	25,44	2,93	0,00773	8,80	10,50	1,19	0,747
S100A8	G0000014354	20,73	56,03	2,70	0,00014	21,05	37,26	1,77	0,238

ctl vs Eex median						ctl vs Aex median			
**gene_name**	**ensembl_id**	**cpm_ctl**	**cpm_case**	**ratio**	**FDR**	**cpm_ctl**	**cpm_case**	**ratio**	**FDR**
CSF3R	G0000011953	7,74	24,45	3,16	0,02079	7,89	9,24	1,17	0,767

ctl vs Eex upper						ctl vs Aex upper			
**gene_name**	**ensembl_id**	**cpm_ctl**	**cpm_case**	**ratio**	**FDR**	**cpm_ctl**	**cpm_case**	**ratio**	**FDR**
GPR97	G0000018288	2,56	13,31	5,20	0,02805	2,62	2,97	1,13	0,871
ITGAX	G0000014067	9,44	25,17	2,67	0,04854	9,74	10,16	1,04	0,968
HCAR3	G0000025539	11,04	27,83	2,52	0,04379	11,42	12,65	1,11	0,791

Top DEGs unique to the A sub-phenotype

ctl vs A median						ctl vs E median			
**gene_name**	**ensembl_id**	**cpm_ctl**	**cpm_case**	**ratio**	**FDR**	**cpm_ctl**	**cpm_case**	**ratio**	**FDR**
Metazoa_SRP	G0000026642	20,22	71,16	3,52	0,00977	19,76	42,73	2,16	0,109
RN7SL1	G0000025848	20,22	70,83	3,50	0,01000	19,76	42,49	2,15	0,118
RN7SL2	G0000026515	34,67	115,33	3,33	0,01497	33,90	81,95	2,42	0,120
RNA45S5	G0000022584	231,86	595,30	2,57	0,03197	225,84	375,97	1,66	0,205

clt vs A upper						clt vs E upper			
**gene_name**	**ensembl_id**	**cpm_ctl**	**cpm_case**	**ratio**	**FDR**	**cpm_ctl**	**cpm_case**	**ratio**	**FDR**
KRT13	G0000017140	9,90	47,72	4,82	0,0045	9,74	15,09	1,55	0,312
SPP1	G0000011878	16,42	45,83	2,79	0,0300	16,14	30,54	1,89	0,062

ctl vs Aex median						ctl vs Eex median			
**gene_name**	**ensembl_id**	**cpm_ctl**	**cpm_case**	**ratio**	**FDR**	**cpm_ctl**	**cpm_case**	**ratio**	**FDR**
TNC	G0000004198	37,74	97,25	2,58	0,03118	37,15	53,00	1,43	0,315

ctl vs Aex upper						ctl vs Eex upper			
**gene_name**	**ensembl_id**	**cpm_ctl**	**cpm_case**	**ratio**	**FDR**	**cpm_ctl**	**cpm_case**	**ratio**	**FDR**
CD36	G0000013521	10,88	36,27	3,33	0,0467	10,60	9,03	0,85	0,920

Given are the unique DEGs with a >2.5-fold increase in expression in either E and A sub-phenotypes of COPD (see Table S4C and S5 for further detail)

E = emphysema-dominant, A= airway disease-dominant, Eex = emphysema-dominant with the strongest CT phenotype,
Aex= airway disease-dominant with the strongest CT phenotype, upper= upper quartile

Expression values (cpm) might be slightly shifted for the control group due to independent normalizations with the edgeR TMM method for the control versus airway and the controls vs emphysema datasets

Table S4E female

Table S4E Top differential genes unique to E and A sub-phenotypes of female COPD									
**ctl vs E upper**						**ctl vs A upper**			
**gene_name**	**ensembl_id**	**cpm_ctl**	**cpm_case**	**ratio**	**FDR**	**cpm_ctl**	**cpm_case**	**ratio**	**FDR**
FGFBP1	G0000013744	11,55	44,12	3,82	1,71E-08	11,58	32,32	2,79	0,06
TPSB2	G0000019725	19,49	58,64	3,01	0,04356	19,54	22,04	1,13	0,93
CDC20B	G0000016428	26,30	68,74	2,61	0,00451	26,37	38,17	1,45	0,27
ANKRD18A	G0000018007	11,17	28,90	2,59	0,02803	11,20	18,83	1,68	0,20
**ctl vs Eex median**						**ctl vs Aex median**			
**gene_name**	**ensembl_id**	**cpm_ctl**	**cpm_case**	**ratio**	**FDR**	**cpm_ctl**	**cpm_case**	**ratio**	**FDR**
CPA3	G0000016375	10,01	60,80	6,08	0,01515	10,11	18,00	1,78	0,67
**ctl vs Eex upper**						**ctl vs Aex upper**			
**gene_name**	**ensembl_id**	**cpm_ctl**	**cpm_case**	**ratio**	**FDR**	**cpm_ctl**	**cpm_case**	**ratio**	**FDR**
TPSAB1	G0000017223	32,77	115,12	3,51	0,00134	33,37	57,75	1,73	0,72
FKBP5	G0000009606	45,85	149,93	3,27	4,32E-06	45,84	51,23	1,12	0,82
SERPINB2	G0000019763	20,44	51,94	2,54	4,28E-06	20,64	34,95	1,69	0,48
DEGs unique to the A sub-phenotype									
**ctl vs A lower**									
**gene_name**	**ensembl_id**	**cpm_ctl**	**cpm_case**	**ratio**	**FDR**	**cpm_ctl**	**cpm_case**	**ratio**	**FDR**
BST2	G0000013030	18,00	36,80	2,04	0,04016	17,95	20,70	1,15	0,70

Given are the unique DEGs with a >2.5-fold increase in expression in either E and A sub-phenotypes of COPD (see Table S4C and S5 for further detail)

E = emphysema-dominant, A= airway disease-dominant, Eex = emphysema-dominant with the strongest CT phenotype,
Aex= airway disease-dominant with the strongest CT phenotype, upper= upper quartile, lower = lower quartile

Expression values (cpm) might be slightly shifted for the control group due to independent normalizations with the edgeR TMM method for the control versus airway and the controls vs emphysema datasets

Table S5 Gprofiler biological process enrichment for cases and E A sub-phenotypes

see separate excel attachment

Table S6 all mitochondrial genes male Eex

see separate excel attachment

**Tables S7 PCR primers and products**			
**RT-PCR**			
CA12	fwd	TGG CAT TCT TGG CAT CTG TAT TG	109 bp
	rev	CTT GGT GGC TGG CTT GTA AAT G	
source	Scrideli et al., J. Neurooncol. Vol. 88, p281, 2008		
CEACAM5	fwd	CTG TCC ACC AAG ATC AAG CAG	236 bp
	rev	AGC GAC CAC ATA GGG AGA AAA T	
source	selected using Primer3 software		
C8orf4	fwd	CAT GCC CTG TGC TGT TAC AGA	164 bp
	rev	AAG TGA CCC TGG ATA AAT GTC AT	
source	Zhang et al., Med Oncol, Vol 28, S647, 2011		
HBB	fwd	TCT GTC CAC TCC TGA TGC TGT	239 bp
	rev	GCA CTG GTG GGG TGA ATT CTT	
source	selected using Primer3 software		
MT-CO2	fwd	ACT GAA CCT ACG AGT ACA CCG A	323 bp
	rev	TTA ATT CTA GGA CGA TGG GCA TG	
source	Abriel et al., The Prostate, Vol. 68, p1086, 2008		
SELL	fwd	AAA ACC CAT GAA CTG GCA AAG G	219 bp
	rev	TCA CCA TCT CCC CAG TTC TCT	
source	selected using Primer3 software		
CXCL1	fwd	GCG CCC AAA CCG AAG TCA TA	213 bp
	rev	CCT CTG CAG CTG TGT CTC TC	
source	selected using Primer3 software		
a-enolase	fwd	GAG CTC CGG GAC AAT GAT AAG	159 bp
	rev	TGT TCC ATC CAT CTC GAT CAT C	
source	selected using Primer3 software		
**genomic PCR**			
MT-CO2	fwd	CCC CAC ATT AGG CTT AAA AAC AGA	80bp
MT-CO2	rev	TAT ACC CCC GGT CGT GTA GCG GT	
SDHA	fwd	TCT CCA GTG GCC AAC AGT GTT	71bp
SDHA	rev	GCC CTC TTG TTC CCA TCA AC	
source	Thesis M.L. Fuentes, Quantification of mitochondrial DNA in		
	human whole blood using real-time qPCR, Universidad Zaragoza,		
	Spain, 2012		
	http://zaguan.unizar.es/record/9055		

Supplement: Deconvolution analysis for leukocyte populations

We applied our gene expression data to the xCell analysis (Aran et al. Genome Biology (2017) 18:220) and we checked for neutrophils, macrophages and mast cells in male and female E and A samples.

This analysis revealed no significant difference for male and female neutrophils in E, a trend for macrophages in male A (p=0.053) but not female A (p= 0.249). Conversely, xCell showed a trend for mast cells in female E (p=0.062) but not male E (p=0.426). Of note M2 macrophages were found significant for male A (p= 0.031) but not for female A (p= 0.227).

Table S8 xCELL deconvolution analysis of EvA transcriptome E vs A

males	greater in A	greater in E
**macrophages**	**0.053**	0.948
**M1**	0.218	0.784
**M2**	**0.031**	0.969
**mast cells**	0.577	0.426
**neutrophils**	0.837	0.165

females	greater in A	greater in E
**macrophages**	0.249	0.758
**M1**	0.505	0.505
**M2**	0.227	0.78
**mast cells**	0.941	**0.062**
**neutrophils**	0.903	0.101

shown are p-values in Wilcoxon Whitney U test

Supplement: Method RNA sequencing and data processing methods

RNAseq was performed in that indexed cDNA libraries were prepared on Sciclone robot (Perkin Elmer Inc., MA, USA) using the RNATruSeq protocol (RS-122-2001 and RS-122-2002, Illumina Inc., C.A. USA). Average size of the PCR products purified on AMPure XP beads (Beckman Coulter, Inc, Fullerton, CA USA) was 325±30bp. The paired-end 100bp read sequencing of the transcriptome was performed on pools of 6 cDNA libraries using Hiseq 2000. Additional sequence was generated when coverage was not sufficient. Sequencing yields were at 5.3±1.6Gb.

All FASTQ files produced from sequencing of RNAseq samples were aligned to a human reference genome using CASAVA software version 1.7 to assess sequence quality. Bronchial brushing and bronchoalveolar lavage samples coming from the same patient were compared based on SNPs called from their RNAseq data in order to ensure proper assignment. Next we selected the samples based on the number of reads (a minimum of 10M paired-end reads mapped to genes) and the average quality in order to avoid biases due to sample pooling.

The selected RNAseq samples were then aligned with the GEMTools RNAseq pipeline v1.7 (http://gemtools.github.io), which is based on the GEM mapper [1]. The pipeline aligns all reads in a sample

in three phases, mapping against the reference genome, against a reference transcriptome, and against a de-novo-transcriptome, which was generated from the input data to detect new junction sites. The transcriptome was generated from version 15 of the Gencode annotation. After mapping, all alignments were filtered to increases the number of uniquely mapping reads. The filter criteria contained a minimum intron length of 20, a minimum exon overlap of 5 and a filter step against the reference annotation checking for consistent pairs and junctions where both sides align to the same annotated gene. Quantifications and read counts were calculated using the Flux Capacitor [2]. This tool quantifies RNAseq reads on isoform level and the resulting counts were summed up to create gene-level read counts that were used for the differential expression analysis.

Supplement: LASSO method

For LASSO regression the glmnet R package with family=‘binomial‘ was used. The input expression dataset contained the 40 DE genes between control and cases for males and the 73 DE genes between control and cases for females. Cross-confirmation was performed with controls and cases with CT scan. For prediction (male dataset), 36 controls and 36 cases without CT scan were used. We run 100 iterations each time randomly selecting 36 controls for prediction and taking out these 36 controls from the cross-confirmation. The predictions were done with lambda 1 sd, For the female dataset, the same methodology was applied but with 17 no CT cases and 17 randomly selected controls for prediction. The negative and positive predictive values were calculated for each iteration and then averaged for all iterations. Genes with coefficients >0 and appearing >= 90 times out of 100 iterations were selected in the male dataset for plotting. For the female dataset, genes with coefficient >0 and appearing >= 65 were selected for plotting.

Supplement: RT-PCR Method

RNA isolated using the AllPrep DNA/RNA Mini Kit (#80204, Qiagen, Hilden, Germany and 100 ng RNA was reverse-transcribed using MuLV Reverse Transcriptase (#N808-0018, Thermo Fisher Scientific), RNase Inhibitor (#N808-0019, Thermo Fisher Scientific), and oligo(dT) (N808-0128, Thermo Fisher Scientific) as primer according to the manufacturer’s instructions.

Using the LightCycler 2 system (Roche Diagnostics, Mannheim, Germany), semiquantitative PCR was performed with primers given in Table S7. In brief, 2µl cDNA was used with the Fast Start SYBR Green I Master Mix (#04707516001, Roche) with the following settings: denaturation for 10 min at 95°C; 40 cycles of annealing for 10 sec at 60°C, elongation for 25 sec at 72°C, melting for 5 sec at 95°C; and a final melting curve from 65 to 95°C with an increase of 0.2°C/sec. Performing genomic DNA for PCR, 9 ng DNA was used with the following settings: denaturation for 10 min at 95°C; 50 cycles of annealing for 5 sec at 58°C, elongation for 5 sec at 72°C, melting for 10 sec at 95°C; and a final melting curve from 65 to 95°C with an increase of 0.2°C/sec.

For analysis, all samples are processed in the Light-Cycler software (Roche, Penzberg, Germany) with the same settings including the same thresholds. Cycle number of the target gene was subtracted from the corresponding alpha-enolase housekeeping gene, and its value was subsequently calculated to the power of 2.

Supplement: Method Cell culture and stimulation
16HBE14o- cells (human bronchial epithelial cell line kindly provided by Dieter C. Gruenert from the University of California, San Francisco, CA, USA, Cozens, A.L.*, et al.* CFTR expression and chloride secretion in polarized immortal human bronchial epithelial cells. *Am J Respir Cell Mol Biol* 10, 38-47 (1994)) were treated with H2O2 (# H1009, Sigma-Aldrich) for 4 hs, cells were harvested by using trypsin-EDTA buffer (Sigma #T4049) and RNA was isolated from Tri-reagent (Sigma #T9424) lysates using chloroform extraction according to manufacturer's instruction and RNA reverse transcribed and amplified as above. RNA was isolated and RT-PCR for CXCL1 versus alpha-enolase was performed. Results are normalized to alpha enolase and expressed as fold increase over untreated cells, n=3, *p<0.05, Mann-Whitney U Test.

<u>Supplement: Analysis of differential genes versus controls in male and female COPD.</u>

For males the gene expression levels for the 40 genes in the individual samples are illustrated in Figure S1A and this indicates that many cases show a strong increase of the upregulated genes and simultaneously a strong decrease of the down-regulated genes. For confirmation we analysed a separate group of n=36 male COPD patients, who were also recruited under EvA but without CT data and who were not included in the discovery group of n=173 (see Table S1). The cases from the confirmation group (blue top bar in figure 1A) also showed the same up- and downregulation pattern of the respective genes.

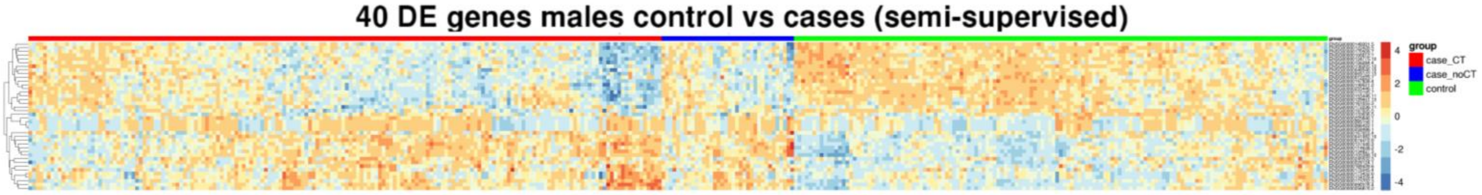

Figure S1A: semi-supervised hierarchical clustering of the male COPD DEGs
Hierarchical clustering: Genes are listed in Table S3 cases and controls were grouped as red: male cases n= 173, green: male controls n= 145, blue: male confirmation group n= 36. The confirmation group consisted of patients, who have not had a chest CT and had not been included in the discovery group of samples.

When using the LASSO approach (least absolute shrinkage and selection operator) to define uncorrelated genes with the strongest predictive value, we found CA12, CCDC81 and SCGB1A1 as top predictors and these were able to separate cases and controls (Fig S1C). Here the confirmation cases (blue) were congruent with the discovery group of cases.

For females the levels of gene expression for the 73 genes in the individual samples is illustrated in Figure S1B and this indicates that many cases show a strong increase of the upregulated genes and simultaneously a clear decrease of the down-regulated genes when compared to controls. For confirmation we analysed a separate group of n=17 female COPD patients, who were also recruited under EvA but without CT data and who were not included in the discovery group of n=86 (see Table S1). The cases from the confirmation group (blue top bar in Figure S1B) also showed the same pattern of up- and downregulation of the respective genes.

We did not obtain a significant p-value (Spearman corr=0.01, p-value =0.27 for the rank preservation test between DEGs for male and female COPD. Hence, the pattern of DEGs is clearly different between males and female also by this approach.

Of note, CYP1B1 is one of the top DEGs in female COPD. While this cytochrome is involved in metabolism of steroids we found no association of glucocorticoid use (51.5% of females on inhaled glucocorticoids) and CYP1B1 expression (p=0.39, Wilcoxon rank test).

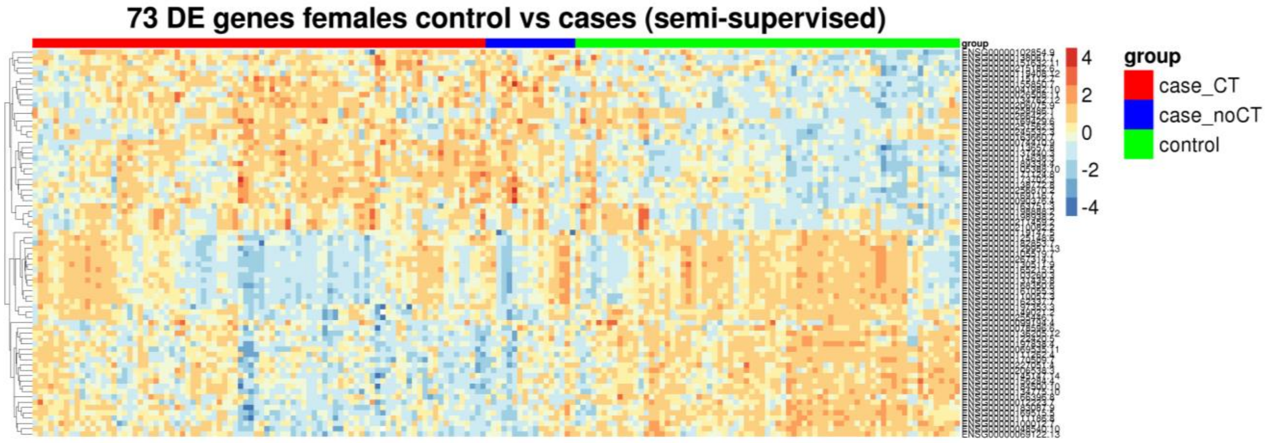

Figure S1B: semi-supervised hierarchical clustering of the female COPD DEGs

Hierarchical clustering: Genes are listed in Table S3 cases and controls were grouped as red: female cases n= 86, green: female controls n= 73, blue: female confirmation group n= 17. The confirmation group consisted of patients, who have not had a chest CT and had not been included in discovery group of samples. Figure S1A, B was produced using the R package pheatmap, pheatmap: Pretty heatmaps [Software] R Kolde, URL https://CRAN. R-project. org/package= pheatmap.

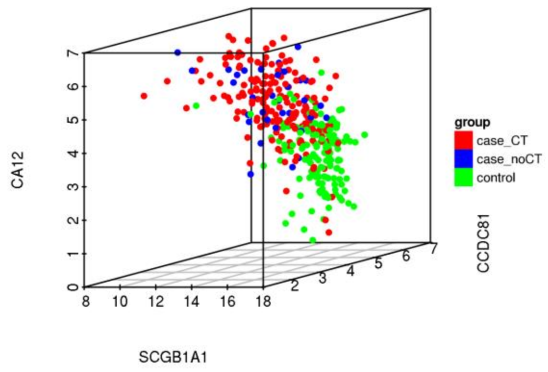

Figure S1C: 3D plot of top 3 LASSO DEGs in males

After running 100 lasso iterations, each time with a different randomization of controls, the 3 top genes with coefficient different from 0 appearing more times were selected: CA12 =99/100, CCDC81 =93/100, SCGB1A1 = 90/100. The median PPV (positive predictive value) was 0.80 +/-sd 0.13 and the median NPV (negative predictive value) was 0.83+/- 0.07. . red = cases green = controls, blue = confirmation group

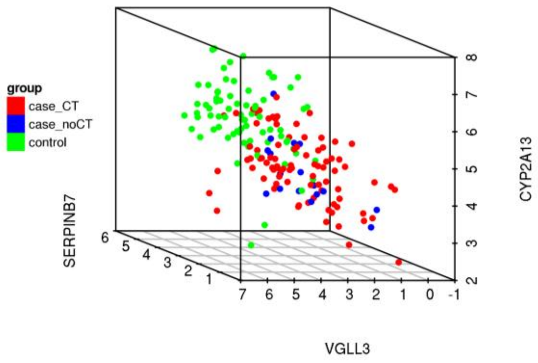

Figure S1D: 3D plot of top 3 LASSO DEGs in females

LASSO 3D plot: After running 100 lasso iterations, each time with a different randomization of controls, the 3 top genes with coefficient different from 0 appearing more times were selected: SERPINB7 = 86/100, VGLL3 =75/100 and CYP2AB =65/100. The median PPV (positive predictive value) was 0.80+/- 0.09 and the median NPV (negative predictive value) was 0.80+/- 0.07. red = cases green = controls, blue = confirmation group.

When using the LASSO approach to define genes with the strongest uncorrelated predictive value for the female DEGS, we found Serpin B7, VGLL3 and CYP2A13 as top 3 predictors and these were able to clearly separate cases and controls (Fig S1D). Here the confirmation cases (blue) showed a pattern similar to the discovery group of cases.

<u>Supplement: RNAseq transcript levels for CEACAM5 and CA12 for males and females and RT-PCR confirmation of gene expression data in COPD</u>

We then took a closer look at the genes with increased expression in COPD and analysed the top genes shared between males and females, i.e. CA12 and CEACAM5. For CA12 the RNAseq expression values in females were slightly lower but the increase of the median in COPD compared to controls was similar in both males and females at around 3-fold (see Figure S2A). A similar pattern was seen for the expression of CEACAM5 (p<0.0001, for all case control comparisons). In the separate confirmation group of patients, we confirmed the significantly higher levels compared to controls for CA12 and CEACAM5 in both males and females (blue boxes in Figure S2A). For experimental confirmation of the RNAseq expression pattern, we used RT-PCR on the RNA of the very same male samples that had been subjected to RNA sequencing. As shown in the supplementary material in Figure S2B there was a positive correlation between the two types of assessment both for CA12 and for CEACAM5.

S2A

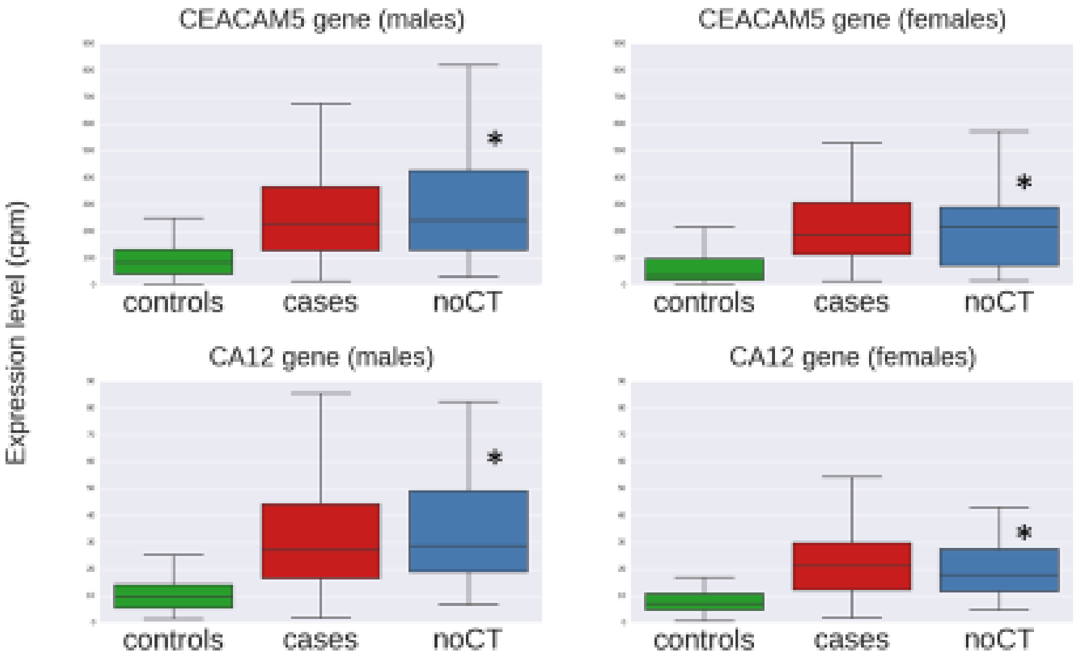

S2B

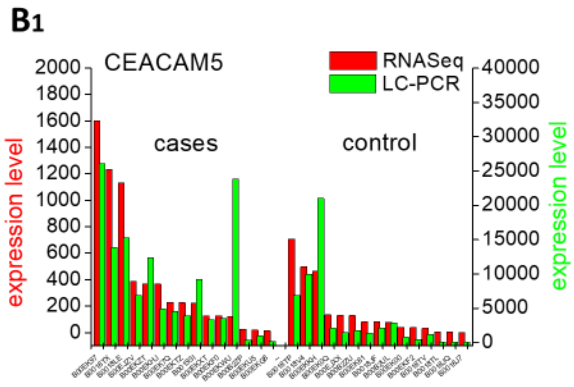

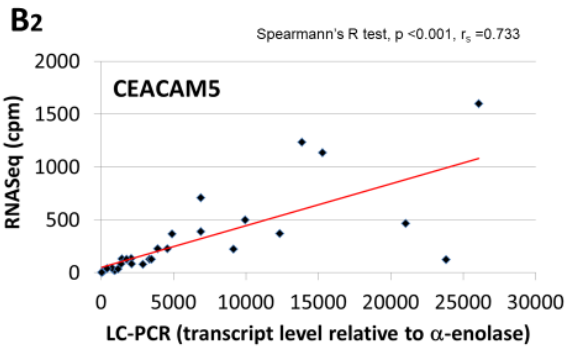

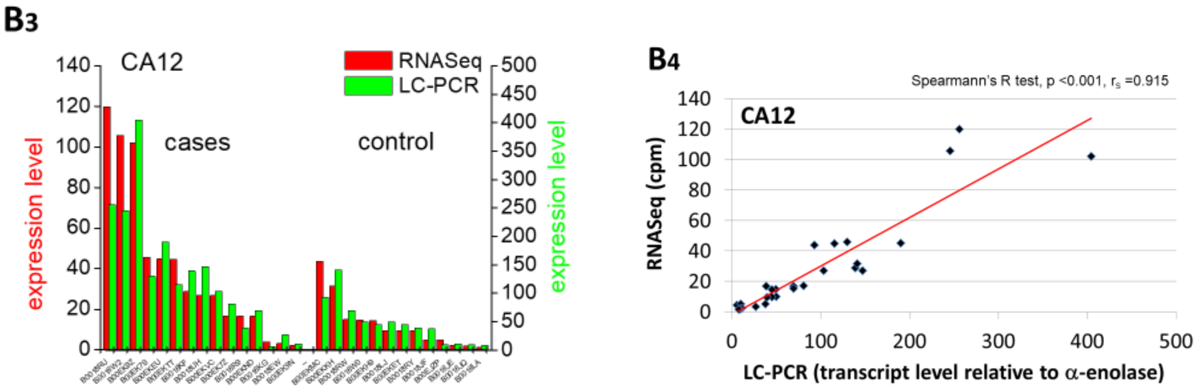

Figure S 2 CEACAM5 and CA12 expression and confirmation of RNAseq expression levels by RT-PCR.

A Shown is the expression level in cpm for male and female controls (green) and COPD case (red) and COPD cases without CT data used for confirmation (noCT, blue). Values in the confirmation group are for males (n= 36) CEACAM5: median 240,4 cpm, CA12: median 28,6 cpm; and for females (n= 17) CEACAM5: median 217,5 cpm, CA12: median 17,8 cpm, * = p <0.0001 compared to control, Wilcoxon rank sum test. The figure was produced using the R package ggplot2 (Wickham H (2016). *ggplot2: Elegant Graphics for Data Analysis*. Springer-Verlag New York. ISBN 978-3-319-24277-4, https://ggplot2.tidyverse.org).

B (1-4) Samples from cases and controls that had been used for RNA sequencing analysis were used for reverse transcription and PCR amplification using primers given in Table S2. Expression levels for CEACAM5 (A,B) and for CA12 (C,D) were normalized to the levels of alpha-enolase. Spearmann's R test, , p <0.001, r_s =0.733 for CEACAM5, p <0.001, r_s =0.915 for CA12.

Supplement comparison to Steiling et al [3]

Among the males 20 and among the females 22 genes were also found by Steiling et al ([3]) and there was an overlap of altogether 27 genes (see Figure S3). Of note there are substantial differences in the patient cohorts in that the EvA patients contained no current smokers and current smoking may induce additional inflammatory genes.

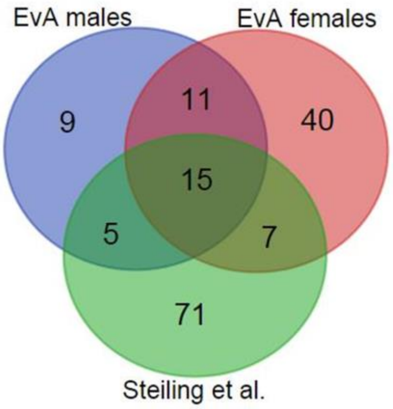

Figure S3 Venn diagram for bronchial brush genes as detected in the EvA study compared to the 98 genes of Steiling *et al.* ([3]) Shown are the EvA genes for males and females separately. This is compared to the genes published by Steiling et la ([3]).

When analyzing our data set with males and females combined, i.e. comparing 259 cases to 265 controls then we identify 41 differential genes. Of these, 22 genes were also found by Steiling et al [3] (see Table S3). Hence, also in the combined male female analysis we detect 19 additional differential genes. Conversely, Steiling et al found 76 DEGs not detected in our combined analysis (see Table S3)

<u>Supplement:</u> <u>Strategy for identification of DEGs unique for emphysema-dominant and airway disease dominant COPD in male brush samples</u>
We compared gene expression in controls (n= 145) to the sub-phenotypes using permutation statistics. With a cut-off of at least a 1.5 fold change of the median expression level we detected 45 DEGs with a significantly different median for controls versus E and 92 DE genes for control versus A (see Table S4A). When we subtract the genes in common for controls versus E and controls versus A at the median (Table S4B) then we have at the median 4 DEGs unique for controls versus E and 21 DEGs unique for controls versus A (Table S4C ctl vs E and A male female unique DE genes, column M) and here the delta case-control is at least 1.5-fold higher in E compared to A. The same approach was applied to the analysis at the lower and upper quartile in order to detect additional genes, which are differentially down or up-regulated in a subset of patients. When combining all of these genes then we found a total of 15 unique DEGs for E and 43 unique DEGs for A (Table S4C ctl vs E and A male female unique DE genes, column AK).
Also, we compared gene expression in controls to the Eex and Aex sub-phenotypes. While this reduces the power by 50% it has the potential to identify additional genes because of the more pronounced and more specific pathology in these cases. Here we found at the median 18 unique DE genes for Eex and 17 unique DE genes for Aex (see Table S4C, column AZ). Combining DEGs identified at lower quartile, upper quartile and median for the extreme samples we find a total of 43 DEGs for Eex and of 54 DEGs for Aex (column BX, Table S4C) and this includes 35 additional genes found for Eex and not for E and 47 additional genes found for Aex and not for to A. These data illustrate that the analysis of the extreme phenotypes generates additional informative genes that are characteristic of sub-phenotypes of COPD.

When combining all of the data then we find at total <u>of 51</u> unique DEGs for controls versus E and Eex (Column CB, Table S4C) and of these 39 DEGs were upregulated (Column CE, Table S4C). For controls versus A and Aex there was a total of <u>90 unique</u> DEGs with 50 upregulated in cases.

Also in females, 18 DEGs were detected only with the Eex approach and 10 only with the Aex approach demonstrating that the analysis of the extreme phenotypes can lead to detection of additional genes also in females.

This study has focused on the impact of COPD and its sub-phenotypes on the airway transcriptome in males and females. Differences in airway gene expression in healthy males versus females have not been analyzed at this point but are likely to exist [4].

Additional variables like age and packyears may impact on the DEGs in COPD and it sub-phenotypes, but our analysis did not detect for high and low age and packyears a difference in expression of top DEGs DEGs CA12, MT-CO2, CSF3R, CD36, CPA3 and BST2 (Wilcoxon rank test, $p>0.05$). Still, a more in depth analysis based on GLM might be able to detect a contribution of these parameters.

<u>Supplement Impact of statistical power</u>:

With the comparison of controls versus E-dominant and controls versus A-dominant cases we have been able to detect 51 DE genes unique for the emphysema-dominant phenotype and 90 DE genes for the airway-disease dominant phenotype in males. For the genes found unique for E we have asked whether there are many among them for which the p-value between controls versus E is just below the cut-off while for control versus A it is just above the cut-off such that an impact of statistical power would be likely.

However, we found only 2 of the 51 E-dominant genes for which the FDR-value for A was less than 10-fold lower in E (CTXN1, IGFBP5) genes marked "y" in Table S4D). Hence, for 49 of the 51 DEGs the significant level of E was more than 10-fold lower compared to the non-significant p value in A. This argues that the difference in power has little impact on the genes identified. Also, the control versus A analysis has revealed also twice as many DE genes compared to controls versus E, in spite of the lower power with only 32 A samples compared to 50 E samples for males.

Likewise, when looking at female samples only 3 of 72 genes unique for E had a less than 10-fold lower p-value in E compared to A, such that in females power appears to have little impact on the DEG discovery as well (Tables S4D).

On the other hand, the failure to detect significant differences by direct comparison of the E and A sub-phenotypes using permutation analysis may be explained by the low statistical power based on the low number of samples with the two CT phenotypes. When analysing data from the perspective of a larger control population we found many genes only differential in comparison to A or only differential in comparison to E. These genes show a differential gene expression pattern between E and A, but here they are not significantly different in direct comparison after correction for multiple testing.

Supplement: <u>DEGs shared by emphysema-dominant and airway disease dominant sub-phenotypes</u>
In males we found n=86 DEGs to be differential for both controls vs E and controls vs A sub-phenotypes (see Table S4B) and of these 29 (34 %, tagged yellow in Table S4B) had also been detected in the case versus control analysis (Table S3). For the 86 DEGs shared by E-dominant and A-dominant cases the GO term "regulation of response to stress" is apparent with altogether 16 DE genes and there is "regulation of response to stimulus" with 25 genes. The latter term includes leukocyte associated genes like FCGR2A, IL8 and CXCR4 (see Table S5).

In females there were 37 genes found for both E and A sub-phenotypes (Table S4B) and 24 (65% tagged yellow in Table S4B) had also been detected in the case versus control analysis (Table S3). The dominant biological process found among the 37 DEGs in common for the female E- and A-dominant COPD cases was "cell migration" (see Table S5).

<u>Supplement: haemoglobin</u>
We have taken a closer look the E-associated top gene haemoglobin-beta (HBB), which showed a 5.2-fold higher median expression level in cases compared to controls. For confirmation of the RNAseq derived gene expression data we performed RT-PCR on the very same RNA samples, which initially had been used for RNA sequencing. Here we found a significant correlation of expression levels between RT-PCR and RNAseq in the airway brush samples (Figure S4).

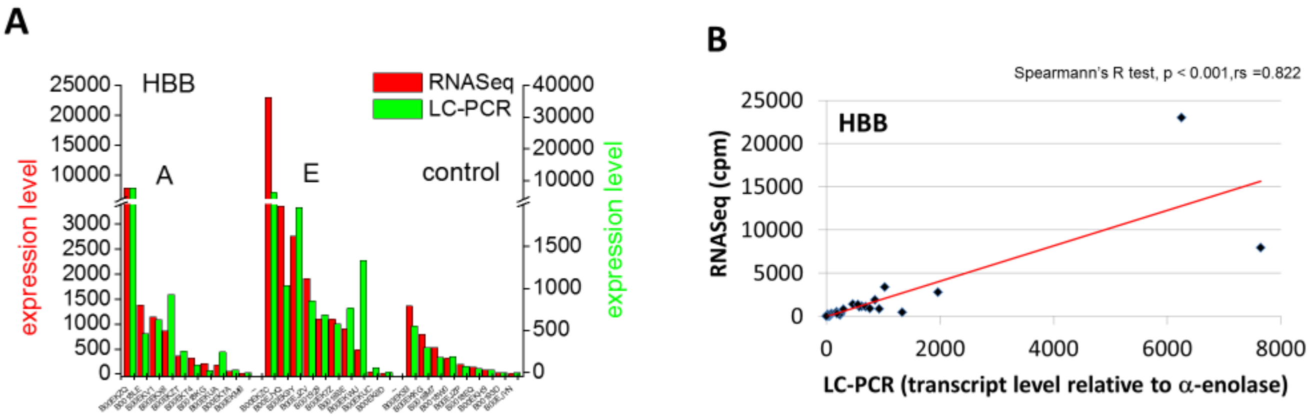

Figure S 4 Confirmation of HBB RNAseq expression levels by RT-PCR.
Samples from controls and case with either E-dominant or A-dominant COPD that had been used for RNA sequencing analysis were used for reverse transcription and PCR amplification using primers given in Table S2. Expression levels for HBB were normalized to the levels of alpha-enolase. (Spearmann's R test, $p < 0.001$,r_s =0.822)

While this upregulation of HBB in bronchial brush samples of E-dominant COPD is surprising, HBB in cells other than the erythrocyte lineage has been reported earlier and this includes macrophages, lens cells, alveolar type II cells, club cells and bronchial epithelial cells [5], [6], [7], [8]. Also we have detected HBB transcripts in the A549 human lung cell line by RT-PCR (data not shown). In our set of cases there was no correlation between bronchial HBB and pO2 level in blood and the role of this gene in the pathophysiology of E-dominant COPD remains to be elucidated.

The finding of HBB expression in lung epithelial cells and cell lines and in bronchial brush samples, supports the notion that HBB is a genuine product of bronchial cells. On the other hand, it is unlikely that the HBB transcripts are derived from contaminating erythrocytes, because only immature red blood cells express low level mRNA and because the red cell marker gene glycophorin A was not among the DEGs in male emphysema in our study.

<u>Supplement: Interaction analysis of DEGs upregulated in male brush samples for COPD sub-phenotypes</u>

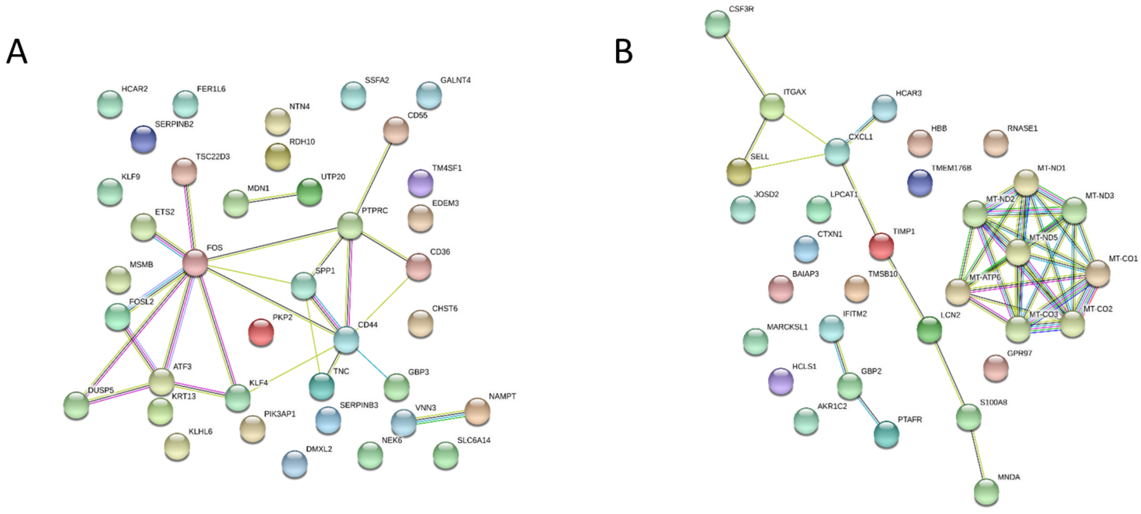

Figure S5 Interaction analysis for genes upregulated in E and A sub-phenotypes of male COPD.
DEGs unique for A-dominant (S5A) and E-dominant (S5B) COPD were analyzed for physical and functional protein interactions were constructed using the string 10.5 database.

<u>Supplement: RT-PCR confirmation of gene expression data for MT-CO2</u>

For confirmation of the RNAseq-derived mitochondrial gene expression data, we performed RT-PCR on the very same RNA samples, which initially had been used for RNA sequencing. For this, we focussed on MT-CO2 and as shown in Figure S6, the pattern for RT-PCR was similar to the results obtained with RNAseq and there was a strong correlation between the two types of gene expression analyses.

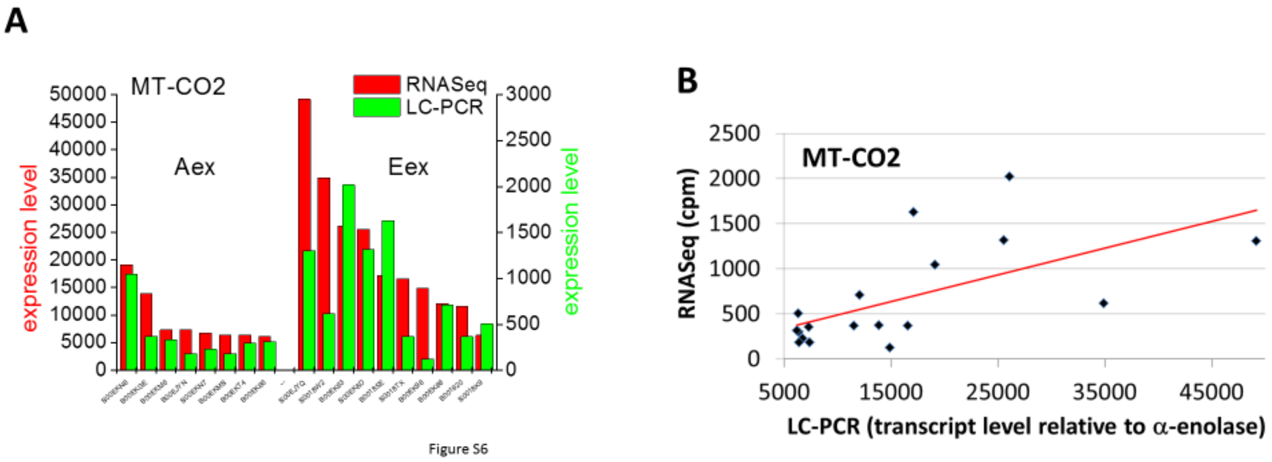

Figure S6 Confirmation of RNAseq expression levels by RT-PCR.
Samples from controls and case with either Eex or Aex COPD that had been used for RNA sequencing analysis were used for reverse transcription and PCR amplification using primers given in Table S2. Expression levels were normalized to the levels of alpha-enolase. (Spearman's test, $p < 0.005$, $r_s = 0.619$)

<u>Supplement: Un-supervised hierarchical clustering of MT-genes, macrophage associated genes and neutrophil associated genes in COPD sub-phenotypes</u>
Genes either selected based on string analysis (MT-genes) or using GO profiler analysis (neutrophil and macrophage associated "stress" genes) where analysed in hierarchical clustering of male samples. Eex

and Aex samples gave a clear pattern (Figure S7), while samples from the noCT confirmation group was not informative (data not shown) Unsupervised hierarchical clustering of Eex and Aex samples revealed a group of samples with low MT genes, which goes along with increased macrophage genes, while high neutrophil genes are associated with high MT genes (see Figure S7).

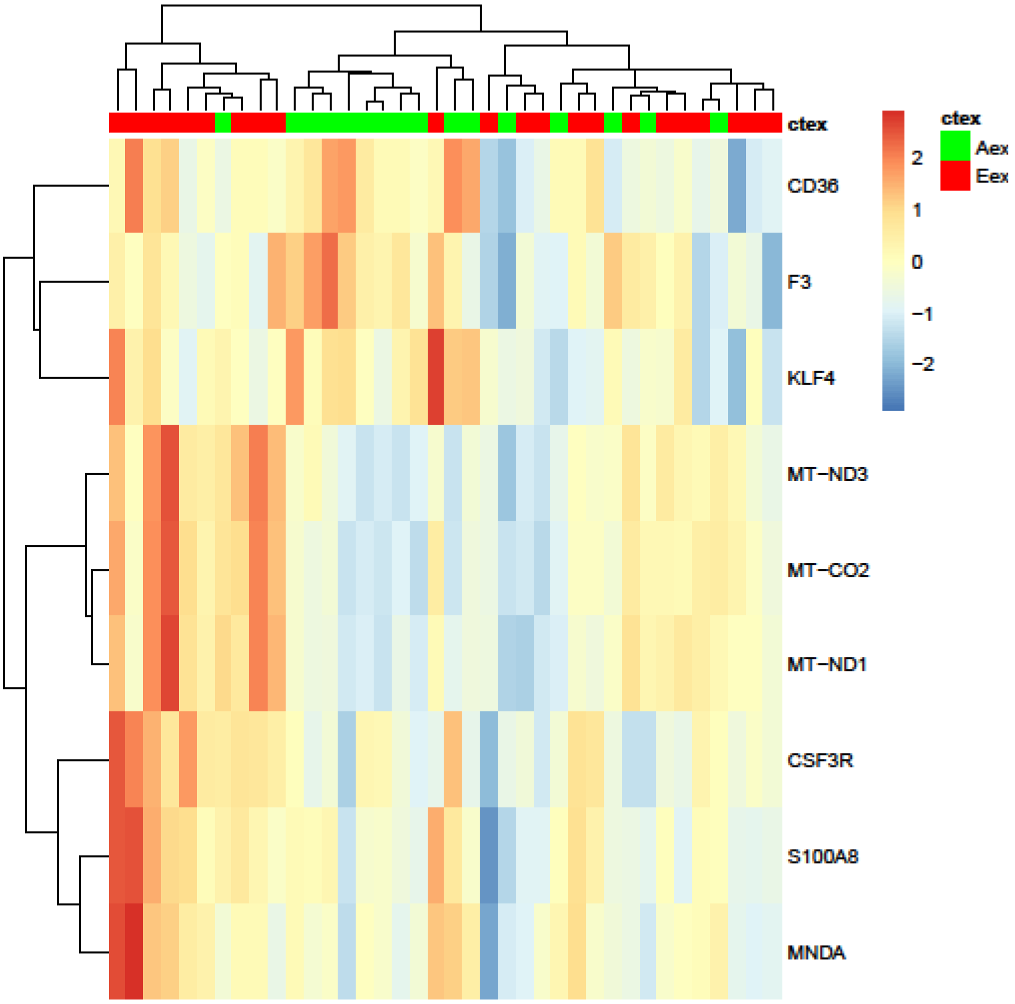

Figure S7 Un-supervised hierarchical clustering of MT-genes, macrophage associated genes and neutrophil associated genes in Eex and Aex COPD sub-phenotypes. A group of samples (mainly Eex) showed high MT-gene levels and high neutrophil associated DEGs CSF3R, S100A8 and MNDA, while a group of mainly Aex samples showed low MT-gene expression levels and high macrophage associated gene expression. The figure was produced using the R package pheatmap, pheatmap: Pretty heatmaps [Software] R Kolde, URL https://CRAN. R-project. org/package= pheatmap.

The association of neutrophil genes and MT genes in these samples (see also Figure 5) suggests that combining these genes might improve the predictive power for molecular identification of emphysema-dominant disease. However, the product of the normalized values for MT-CO2 and CSF3R did not correlate with TLCO/Va in the no CT confirmation group (data not shown). When evaluating the top 5 samples with the highest product of MT-CO2 and CSF3R expression levels then this gave no significant difference (p= 0,178, Mann-Whitney-U-Test) for TLCO/Va, when compared to the bottom 10 samples for this value, quite in contrast to the findings for the set of 8 MT genes alone (see Figure 3C).
A larger cohort of patients will be required to address the question of predictive use of neutrophil genes.

<u>Supplement String interaction analysis of DEGs in female sub-phenotypes</u>

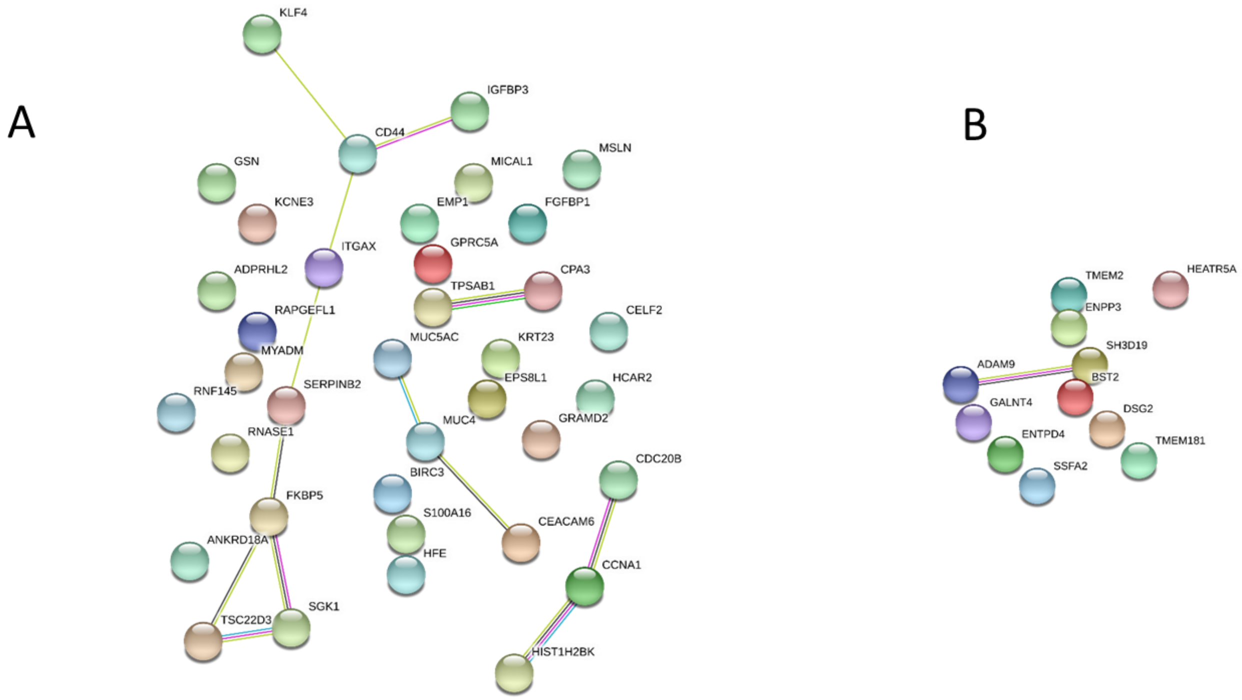

Figure S8 Interaction analysis for genes upregulated in E and A sub-phenotypes of female COPD. DEGs unique for E-dominant (S8A) and A-dominant (S8B) COPD were analyzed for physical and functional protein interactions were constructed using the string 10.5 database.

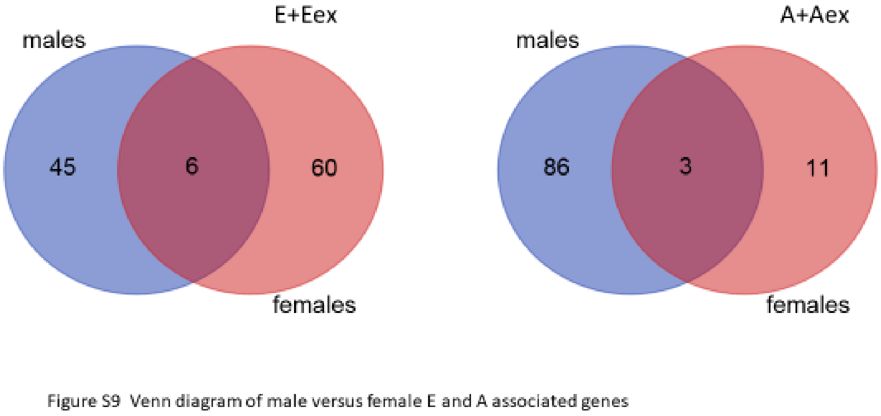

Figure S9 Venn diagram of male versus female E and A associated genes

<u>Supplement: DEGs in mixed and normal CT sub-phenotypes of male COPD</u>

Patients with both high wall area and low lung density in chest CT were defined as mixed cases (see [9]). When comparing gene expression in such cases with a mixed phenotype then we found 14 DEGs (Table S4A) and none of these was unique to either E or A, (Table S4C). Analysis of mixex cases revealed no DE genes.

Furthermore, cases with low wall area and high lung density were defined as normal-CT cases. These formed the largest group of 81 male cases in our cohort. For these we found 119 differential genes for

normal-CT plus normal-CT-ex analysis (Table S4A) and this included 34 genes that had been found to be unique to E- or to A- COPD sub-phenotypes (Table S4C). Looking at the most extreme normal-CT cases, i.e. those clearly separated from cases with abnormal CT phenotype than we picked up 24 of these 34 DEGs unique to either E or A. This indicates that patients with mild disease and normal CT measures of COPD can already express genes, which are characteristic of more advanced dichotomized COPD.

Supplement: DEGs in mixed and normal-CT sub-phenotypes of female COPD

For females we had only 7 mixed cases but we still could detect 38 DE genes (Table S4A) and of these 1 was unique to A and 9 were unique to the E sub-phenotype (Table S4C). Analysis of mixex cases revealed no DE genes.

When looking at normal-CT cases then we found a total of 195 DEGs compared to controls (Table S4A) and of these 51 were also detected among the E- and A-sub-phenotype (Table S4C). Of these 51 genes only 7 were shared with males. Finally, the normal-CT-ex female cases, i.e. those with a clearly normal CT phenotype, also expressed 29 DEGs found to be unique to either E or A (TableS4C) and of these only 2 were shared with males. Taken together similar to males, females with mild disease and normal CT measures of COPD can already express genes, which are characteristic of more advanced dichotomized COPD.

It is, however, a matter of debate as to whether xCell is adequate for this type of analysis, since it covers 64 cell types and excludes genes expressed by carcinoma cells. Among the 64 cell types are - in addition to various immune cells - 23 other cell types including adipocytes, hepatocytes, skeletal muscle cells, chondrocytes, melanocytes and astrocytes. To this set of cells, the following strategy was applied: “For each data source independently we identified genes that are overexpressed in one cell type compared to all other cell types.” (Aran et al. Genome Biology (2017) 18:220). With this approach, typical macrophage genes like CD36 are associated with melanocytes and not with macrophages. Also, typical genes induced in inflammation like IL-2 and TNF are not covered, such that information on cytokines characteristic for T cells and macrophages are not considered. We speculate that an amended version of xCELL, dedicated to leukocytes only, might result in significant results for all of the differential cell types found in our study.

In any event, additional studies, including single cell sequencing, are required to confirm the presence of neutrophils in E-dominant and not in A-dominant disease and the presence of macrophages in A-dominant and not in E-dominant disease in male COPD patients.

References

1. Marco-Sola, S., Sammeth, M., Guigo, R. & Ribeca, P. The GEM mapper: fast, accurate and versatile alignment by filtration. *Nat Methods* **9**, 1185-1188 (2012).
2. Montgomery, S.B.*, et al.* Transcriptome genetics using second generation sequencing in a Caucasian population. *Nature* **464**, 773-777 (2010).
3. Steiling, K.*, et al.* A dynamic bronchial airway gene expression signature of chronic obstructive pulmonary disease and lung function impairment. *Am J Respir Crit Care Med* **187**, 933-942 (2013).
4. Oliva, M.*, et al.* The impact of sex on gene expression across human tissues. *Science* **369**(2020).
5. Liu, L., Zeng, M. & Stamler, J.S. Hemoglobin induction in mouse macrophages. *Proc Natl Acad Sci U S A* **96**, 6643-6647 (1999).
6. Newton, D.A., Rao, K.M., Dluhy, R.A. & Baatz, J.E. Hemoglobin is expressed by alveolar epithelial cells. *J Biol Chem* **281**, 5668-5676 (2006).
7. Skawran, B.*, et al.* Bronchial epithelial cells as a new source for differential transcriptome analysis after lung transplantation. *Eur J Cardiothorac Surg* **36**, 715-721 (2009).
8. Ishikawa, N.*, et al.* Hemoglobin alpha and beta are ubiquitous in the human lung, decline in idiopathic pulmonary fibrosis but not in COPD. *Respir Res* **11**, 123 (2010).
9. Subramanian, D.R.*, et al.* Emphysema- and airway-dominant COPD phenotypes defined by standardised quantitative computed tomography. *Eur Respir J* **48**, 92-103 (2016).
